# Corals regulate the distribution and abundance of Symbiodiniaceae and biomolecules in response to changing water depth and sea surface temperature

**DOI:** 10.1038/s41598-021-81520-0

**Published:** 2021-01-26

**Authors:** Mayandi Sivaguru, Lauren G. Todorov, Carly A. H. Miller, Courtney E. Fouke, Cara M. O. Munro, Kyle W. Fouke, Kaitlyn E. Fouke, Melinda E. Baughman, Bruce W. Fouke

**Affiliations:** 1 Carl R. Woese Institute for Genomic Biology, University of Illinois at Urbana-Champaign, Urbana, IL USA; 2 Carl Zeiss Labs@Location Partner, Carl R. Woese Institute for Genomic Biology, University of Illinois at Urbana-Champaign, Urbana, IL USA; 3 School of Molecular and Cellular Biology, University of Illinois at Urbana-Champaign, Urbana, IL USA; 4 Department of Geology, University of Illinois at Urbana-Champaign, Urbana, IL USA; 5 Department of Biology, Denison University, Granville, OH USA; 6 Department of Ecology and Evolutionary Biology, University of California at Santa Cruz, Santa Cruz, CA USA; 7 Department of Geological Sciences, Jackson School of Geosciences, The University of Texas at Austin, Austin, TX USA; 8 The Eugene Bell Center for Regenerative Biology and Tissue Engineering, Marine Biological Laboratory, Woods Hole, MA USA; 9 Department of Evolution, Ecology and Behavior, University of Illinois at Urbana-Champaign, Urbana, IL USA; 10 Roy J. Carver Biotechnology Center, University of Illinois at Urbana-Champaign, Urbana, IL USA

**Keywords:** Ocean sciences, Marine biology

## Abstract

The Scleractinian corals *Orbicella annularis* and *O. faveolata* have survived by acclimatizing to environmental changes in water depth and sea surface temperature (SST). However, the complex physiological mechanisms by which this is achieved remain only partially understood, limiting the accurate prediction of coral response to future climate change. This study quantitatively tracks spatial and temporal changes in Symbiodiniaceae and biomolecule (chromatophores, calmodulin, carbonic anhydrase and mucus) abundance that are essential to the processes of acclimatization and biomineralization. Decalcified tissues from intact healthy *Orbicella* biopsies, collected across water depths and seasonal SST changes on Curaçao, were analyzed with novel autofluorescence and immunofluorescence histology techniques that included the use of custom antibodies. *O. annularis* at 5 m water depth exhibited decreased Symbiodiniaceae and increased chromatophore abundances, while *O. faveolata* at 12 m water depth exhibited inverse relationships. Analysis of seasonal acclimatization of the *O. faveolata* holobiont in this study, combined with previous reports, suggests that biomolecules are differentially modulated during transition from cooler to warmer SST. Warmer SST was also accompanied by decreased mucus production and decreased Symbiodiniaceae abundance, which is compensated by increased photosynthetic activity enhanced calcification. These interacting processes have facilitated the remarkable resiliency of the corals through geological time.

## Introduction

Tropical and subtropical coral reef ecosystems have long been recognized as sensitive indicators of global climate change and oceanic health^1,2^. Scleractinian corals thrive in nutrient-poor (oligotrophic) tropical and subtropical shallow seawater environments around the world. This is due in large part to symbiotic relationships between the host coral animal, unicellular, photosynthetic dinoflagellates belonging to the family Symbiodiniaceae^3^ and other microorganisms, which together form a tightly integrated community collectively called the *coral holobiont*^4–9^. In order to preserve and better manage coral reef ecosystems, urgency is mounting to establish new analytical approaches capable of deciphering and monitoring the underlying mechanisms by which corals have successfully adapted and survived in the face of rapidly changing ancient and modern marine environmental conditions^10–16^. This work has included analyses of adaptations in coral tissue morphology and colony shape (*morphological plasticity*) as well as shifts in carbon translocation between living coral holobiont cells and the surrounding marine environment (*trophic plasticity)*^17–27^.

However, relatively little is known regarding how the three-dimensional (3D) μm-scale distribution and abundance of Symbiodiniaceae cells and biomolecules (chromatophores, calmodulin, carbonic anhydrase and mucus) vary within structurally intact coral tissue biopsies across changing bathymetry and seasonal SST. It is also not fully understood how these components might change seasonally to influence the formation of high density band (HDB) and low density band (LDB) skeletal layers. As a result, it remains uncertain how coral holobiont tissue cells and biomolecules successfully respond (acclimatize) to changes in water depth (WD) and seasonal sea surface temperature (SST) as is also reflected by overall skeletal structure. This is in large part because previous studies have often relied on techniques that either: (1) physically disrupt the original 3D context of coral holobiont tissue structure, which is destroyed when an air gun or water pick is used to remove and homogenize coral tissues from the skeleton^28–30^; or (2) used fiber optic analyses on bulk coral tissue^31^. Exceptions include three previous studies that have completed analyses of multiple types of cell autofluorescence within the context of original, undisturbed coral tissue structure. For instance, Salih et al.^32^ quantified bulk cellular tissue activity from fluorescent pigments and photo-protective chromatophores in multiple coral species on the Great Barrier Reef. Piggot et al.^33^ quantitatively compared the two-dimensional (2D) abundance of Symbiodiniaceae and mucocyte cells in tissue-skeleton biopsies of *O. annularis* collected on Curaçao from controlled reef tract shading experiments and during seasonal changes in SST. Results identified correlations between seasonal SST and the abundance of Symbiodiniaceae and mucocyte in tissues, indicating that the *O. annularis* coral holobiont transitions from autotrophic to heterotrophic feeding strategies as SST increases. Sivaguru et al.^34^ then qualitatively tracked shifts in the 3D distribution of Symbiodiniaceae and chromatophores in tissues of *O. annularis* and *O. faveolata*, also from Curaçao, during seasonal shifts in carbon translocation.

The present study was conducted to directly test and advance hypotheses developed from these previous studies to investigate the specific cellular and biochemical changes corals make to acclimatize to increasing water depth and seasonal changes in SST. One hypothesis being tested is whether the 3D distribution and abundance of chromatophores can be modified by the coral itself to protect Symbiodiniaceae tissue distribution, abundance and function across bathymetric and seasonal gradients. Another is whether simultaneous changes in the 3D distribution and abundance of coral tissue biomolecules involved in skeletal precipitation might be associated with the formation of skeletal density banding. Next-generation two-photon laser scanning fluorescence microcopy was used to quantify shifts in the 3D distribution of Symbiodiniaceae, chromatophores biomolecules in tissues of *O. annularis* and *O. faveolata*. These analyses included: (1) spectral characterization of Symbiodiniaceae and chromatophores within their original structural context of coral tissue layer growth using single-photon and two-photon wavelengths of light; (2) 3D quantification of the distribution and abundance of Symbiodiniaceae and chromatophores throughout the entire volume of individual polyps in *O. annularis* (at 5 m WD) and *O. faveolata* (at 12 m WD); and (3) 2D quantification of cell distribution and density of Symbiodiniaceae (chlorophyll autofluorescence), chromatophores (autofluorescence), carbonic anhydrase (custom designed antibody), calmodulin (monoclonal antibody), and mucus (wheat germ agglutinin [WGA] stain) in *O. faveolata* at 12 m WD across seasons. Results have established a fundamentally new synthesis that better contextualizes water depth adaptions and acclimatization to seasonal variations in SST. This illustrates the physiological resiliency of corals during reciprocal host to symbiont carbon translocation and is reflected by coral skeletal density banding (CSDB) and their sustained survival in ever changing environments conditions through geological time.

## Materials and methods

### Geobiological setting and the *Orbicella* species complex

**Figure 1:**
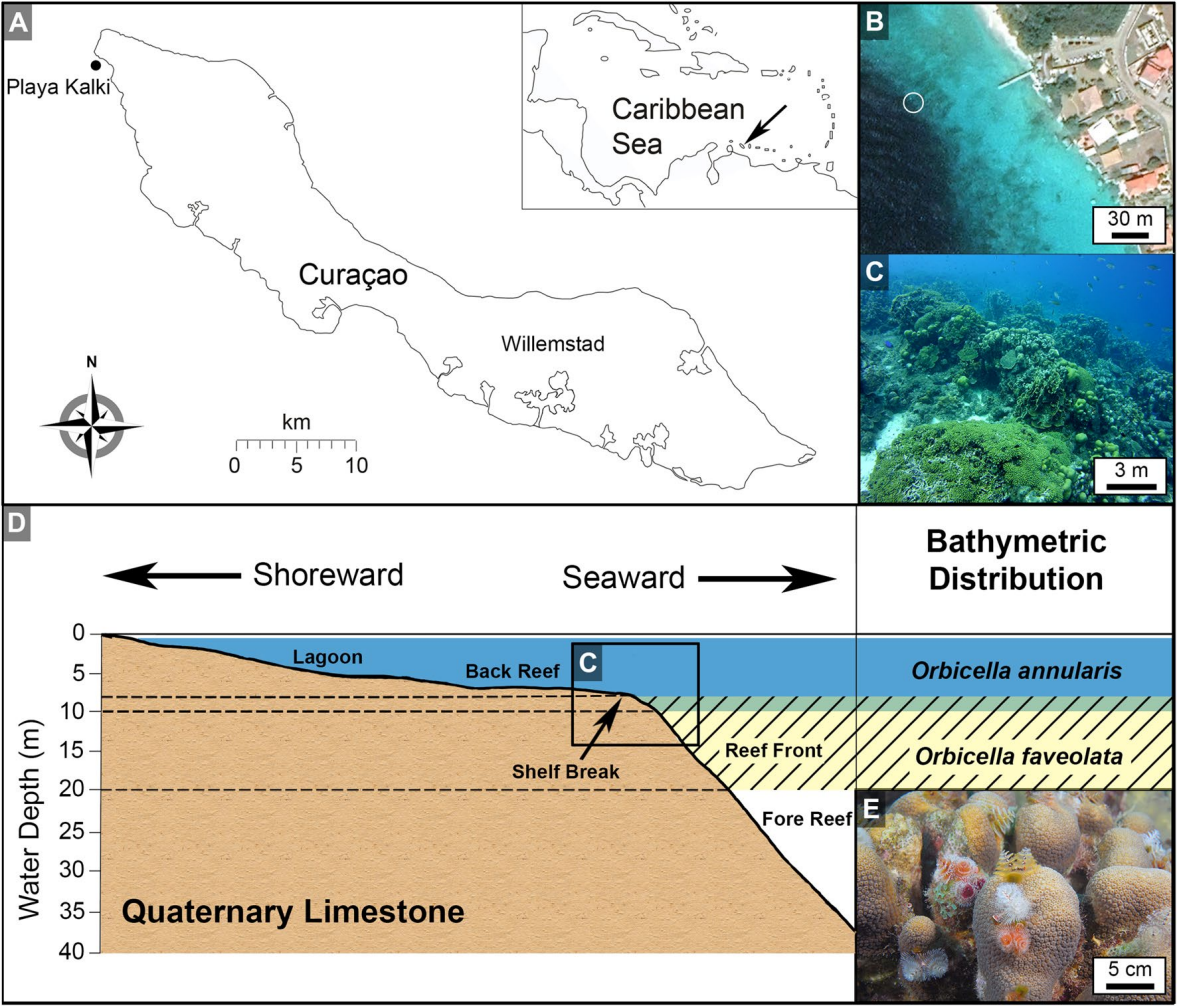
Geographic location and bathymetric distribution of *Orbicella annularis* and *O. faveolata*. (**A**) Map of Playa Kalki on the northwestern end of the island of Curacao in the southern caribbean Sea (see insert). Modified from Fouke et al.^35^ (**B**) Sample site (white circle) where coral biopsies were collected ~100m offshore of Playa Kalki. Image (**B**) was a screen shot from open source Google maps (https://www.google.com/maps/place/Cura%C3%A7ao/@12.1996265,-69.0503172,9152m/). (**C**) Coral constructional buttresses separated by sand channels (spur-and-groove seafloor geomorphology)^36^ on the shelf margin at playa Kalki where coral biopsies were collected. (**D**) Bathymetric cross-sectional profile showing the distribution with respect to the shelf break of the lagoon, back reef, fore reef and reef front marine carbonate depositional facies at Playa Kalki. Modified from Fouke et al.^35^. Area of photograph C shown in black box. *O. annularis* was sampled at 5 m water depth (WD) and *O. faveolata* was sampled at ~ 12 m WD (**E**) Enlargement of small individual columnar heads of *O. annularis with feathery Spriobranchus giganteus* tube-building polychaete borers.

The island of Curaçao lies just north of Venezuela in the southernmost Caribbean Sea (Fig. 1A) and has a 45 km-long continuous fringing coral reef along its leeward coast. This modern-day living reef is deposited atop and adjacent to ancient fossilized Miocene-Holocene coral reef limestones uplifted more than 100 m during transverse motions of the Caribbean and South American crustal plates^35^. These ancient and modern fringing reef ecosystems on Curaçao have been extensively studied^35–37^. In addition, the living fringing reef has been closely monitored and protected by the Caribbean Marine Biodiversity Laboratory (CARMABI) and the government of Curaçao for nearly 75 years. Skeleton-tissue biopsies (2.5 cm-diameter) were collected from apparently healthy colonies of *O. annularis* and *O. faveolata* at Playa Kalki (12° 22′ 31.63″ N, 69° 09′ 29.62″ W; Fig. 1A). Biopsies from both *O. annularis* and *O. faveolata* were collected from 5 to 12 m WD in January 2010. *O. faveolata* biopsies were collected across seasonal changes in SST in March and May 2008 and compared to May 2006 and September 2007 samples reported by Piggot et al.^33^. Annual mean SST on Curaçao in 2001–2010 was 27.5ºC ± 0.5ºC, with seasonal variations of ~ 3 °C that ranged from ~ 26.0 °C in late January to a maximum of ~ 29.0 °C in early September^38^. The reef at Playa Kalki was chosen because the δ^15^N composition of *O. annularis* tissues indicates that it is the least seawater polluted site downstream along the SE-NW flowing offshore current that passes the pollution source of the capital city of Willemstad^39^. The measured photosynthetically active radiation (PAR) at Playa Kalki was 35% at 5 m and 20% at 12 m water depth, indicating ~ 42% reduction in PAR with depth^40^.

**Figure 2:**
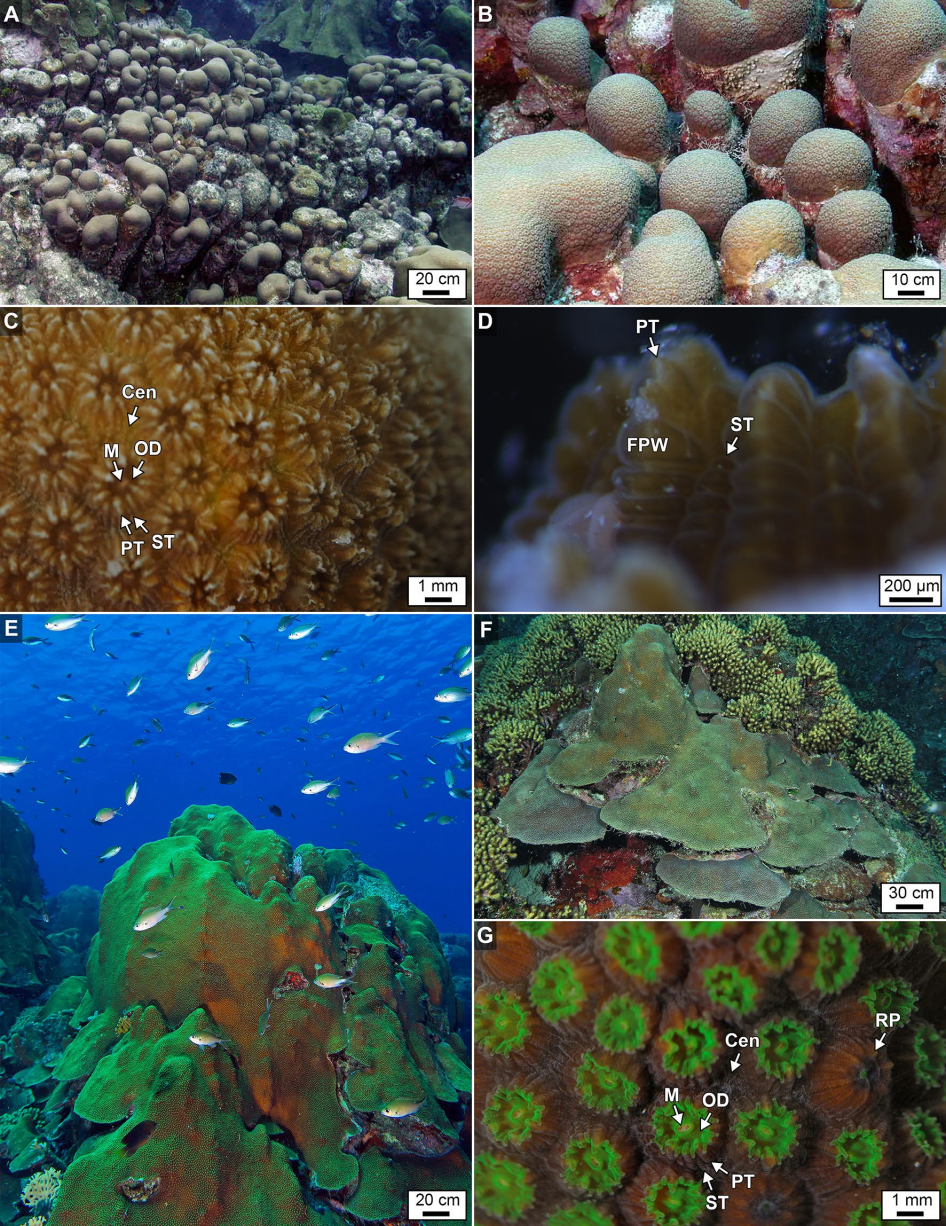
Field and laboratory photographs of *O. annularis and O. faveolata. *(**A**, **B**) Field photographs of *O. annularis *colonies at ~5 m WD at Playa Kalki exhibiting growth of individual columnar heads that are clustered together into an overall hemispherical assemblage. (**C**,** D**) Laboratory photographs of individual *O. annularis* partially opened polyps that are ~2.3 mm in diameter. (**E**,** F**) field photograph of *O. faveolata *colonies at Playa Kalki that grow as massive shapes with knobby ridges that flair out at the lowermost edges of each colony at ~ 12 m WD and broadened morphologies at ~ 18 m WD. (**G**) Laboratory photograph partially opened and partially extened polyps of *O. faveolata *that are ~2.4 mm in diameter. Labels in C, D and G include: Cenceonosarc; FPW_folded polyp wall; PT_ primary tentacle; ST secondary tentacle; OD-oral disk; M-mouth; and RP-retracted polyp.

*O. annularis* and *O. faveolata* were chosen for this study because of their ecological significance as cornerstone framework builders in ancient and modern reefs throughout the Caribbean Sea^36,41,42^. In addition, with respect to the shelf break, these two coral species exhibit different vertical bathymetric distributions with minimal overlap (Fig. 1D)^35,37,43^. Therefore the distribution of *O. annularis* and *O. faveolata* can be used to discriminate between the spur-and-groove back reef and reef front carbonate sedimentary depositional facies in ancient fossilized coral reef limestones (Fig. 1)^35,44,45^. *O. annularis* and *O. faveolata* (Fig. 2), which were originally taxonomically classified as *Montastrea annularis* and *M. faveolata* and later re-assigned^46^, are two closely phylogenetically related scleractinian coral species that are part of the *Orbicella* species complex^41,46,47^. Hermatypic scleractinian corals fundamentally depend upon endosymbiosis with Symbiodiniaceae^4^. To protect the Symbiodiniaceae from photo-inhibition and enhance photosynthetic activity, the coral produces green fluorescent chromatophore cells within the oral ectoderm that overlay and shade Symbiodiniaceae in the oral endoderm^32^. These intimate cellular relationships foster the symbiosis responsible for the ongoing evolutionary and ecological success of corals in shallow marine oligotrophic ecosystems. Symbiodiniaceae provide oxygen as well as glucose, glycerol, and amino acids that are used by the coral to respire and synthesize fats, carbohydrates and calcium carbonate, while the coral provides nutrients (such as nitrogen and phosphorus) and CO_2_ for Symbiodiniaceae photosynthesis as well as a safe refuge^4,5,48,49^.

Morphological characteristics of *O. annularis* (also called the *lobed star coral*) and *O. faveolata* colonies (also called the *mountainous star coral*), chosen for analysis in the present study at Playa Kalki on Curaçao, are consistent with previously published descriptions^17,41,50^ and briefly summarized here. *O. annularis* forms clusters of columns, each of which are generally 5–30 cm in diameter (Figs. 1E; 2A,B). Living coral polyps occur exclusively on the surface of the outermost 5–30 cm terminous of each column, while the lower portions of each column are dead skeleton that are heavily eroded, bored and encrusted. *O. annularis* tissues exhibit a golden-brown to tan color with polyps that are ~ 2.3 mm in diameter and contain 24 radially distributed tentacles (Fig. 2C)^41^. In contrast, *O. faveolata* colonies are characterized by their massive shapes with knobby ridges that flair out at the lowermost edges of each colony (Fig. 2E)^41,50^. With increasing water depth, *O. faveolata* colonies remain massive but become broader and flatten out to increase surface area and light harvesting by the endosymbiotic Symbiodiniaceae (Fig. 2F)^41^. *O. faveolata* tissues are distinctly darker brown with bright green oral disks (Fig. 2G). *O. faveolata* polyps are ~ 2.4 mm in diameter, which is only slightly larger than those in *O. annularis* and contain an equivalent 24 radially distributed tentacles. In addition, the polyps of *O. annularis* are more closely packed and thus are in higher abundance per unit of surface area (Fig. 2C) than those in *O. faveolata* (Fig. 2G)^41^.

### Nomenclature of coral tissue structure

**Figure 3:**
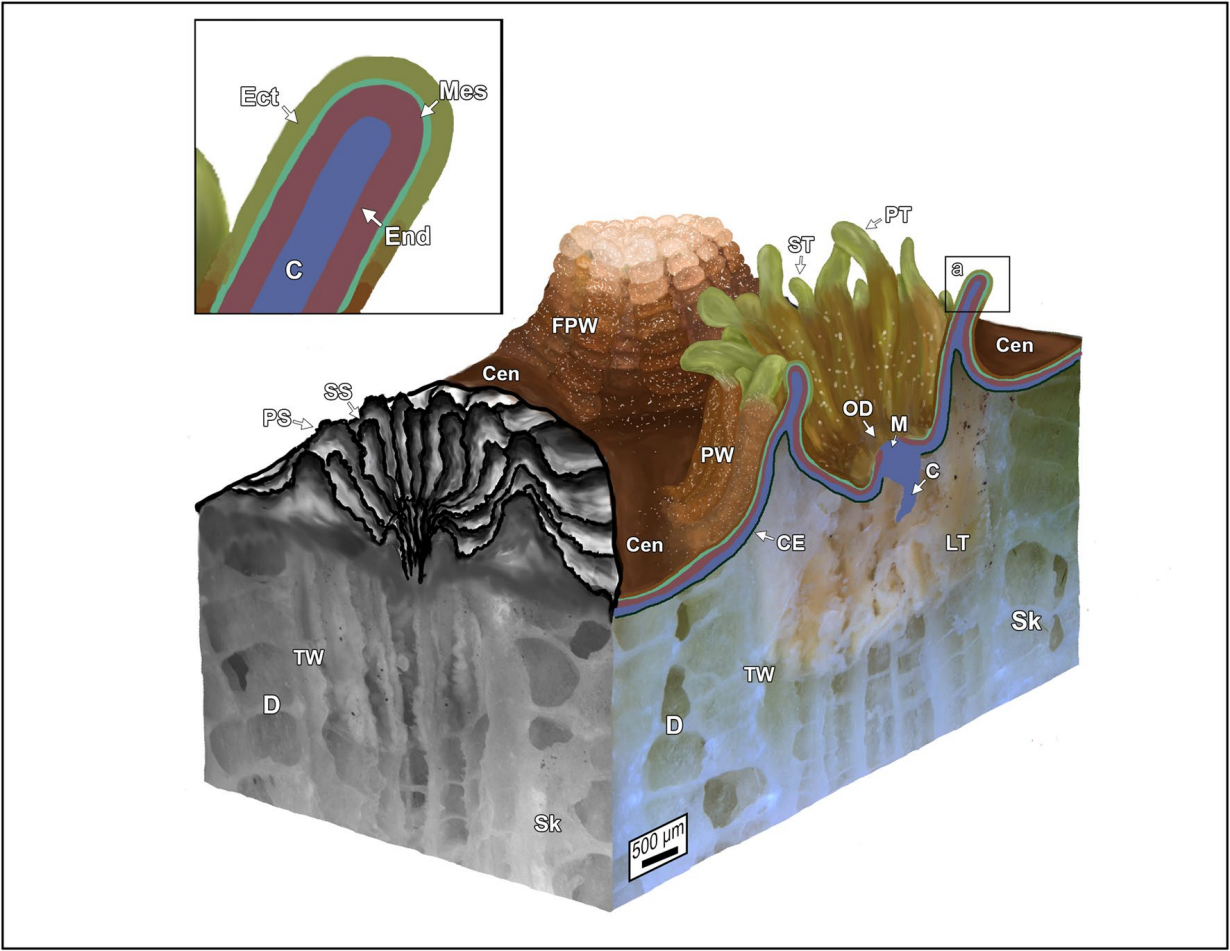
Three-dimensional (3D) block diagram and tissue schematic for *Orbicella annularis* and *O. faveolata.* The sketched uppermost surface of the block diagram shows an extended polyp (right), a retracted polyp (center) and skeletal cup (corallite; left). The vertical side walls of the block are actual photographs of a tissue-skeleton biopsy collected form *O. annularis. *Labels include: Cen–ceonosarc; FPW–folded polyp wall; PT–primary tentacle; ST–secondary tentacle; OD–oral disk; M–mouth; C–coelenteron; CE–calicoblastic epithelium; Sk–skeleton; D–dissepiment; LT–living tissue within the skeleton; TW–thecal wall; PS–primary septum; SS–secondary septum. Schematic enlargement (**a**) illustrates the following basic tissue: C–coelenteron; End–oral endoderm; Mes–mesoglea; and Ect–oral ectoderm. Tissue and skeletal structure terminology after Allemand et al.^54^ and Peters^55^.

Detailed description of the basic anatomy and tissue structure of corals is well-established^48,51–55^. However, the complex terminology used to describe the histological structure of coral tissues has long been challenging and often contradictory^55^. This is in large part because the coral ectoderm changes in composition and function as the animal grows and matures from larval into adult stages and therefore tissue structure nomenclature will depend on the onotogenic stage being studied^55^. As a result, for consistency and clarity in the present study, terminology presented in Allemand et al^54^ will be followed as the primary contextual framework within which the abundance of tissue cells and biomolecules in *O. annularis* and *O. faveolata* is reported. The fundamental living unit of a coral is a cylindrical sac-like polyp that is surrounded by an outer polyp wall that exhibits lobate folds when the polyp is retracted (Figs. 2D, 3)^54^. A central cavity called the coelenteron, which separates the oral and aboral tissues, transports nutrients and gases within and between polyps through the coenosarc^54,56^. The oral tissue of the polyp is in direct contact with seawater and consists of an overlying epidermis layer (oral ectoderm) and an underlying gastrodermis layer (oral endoderm), which are bound together by mesoglea connective tissues (Fig. 3)^54,56^.

Importantly for the present study, the oral endoderm contains photosynthetic endosymbiotic Symbiodiniaceae that are photo-protected by chromatophores in the oral ectoderm^54,56^. The oral ectoderm also contains columnar supporting cells, epitheliomuscular cells, sensory neurons, mucus secreting mucocytes, granule containing pigment cells, stimuli receptor spirocytes and stinging nematocysts. Conversely, aboral tissues at the base of the polyp are composed of an aboral endoderm that is separated by a secondary/aboral mesoglea layer from the underlying calicoblastic epithelium (*calicoblastic ectoderm* or *calicodermis*) layer where the aragonite CaCO_3_ skeleton is precipitated^54,56,57^. Microscopy evidence presented in the present study (see below) indicates that neither *O. annularis* or *O. faveolata* possess an aboral endoderm and secondary/aboral mesoglea, which is contrary to its presence in many other coral pecies^54^.

### SCUBA collection of coral tissue-skeleton biopsies

Sampling and data collection were completed using standard SCUBA techniques. During each dive temperature was measured at sampling depth adjacent to the coral colony using a standard Fisher alcohol glass thermometer, protected in a PVC housing, and a temperature probe built into a ScubaPro dive computer. The dive computer was calibrated against the standard thermometer in the air, underwater, and in seawater prior to each dive. Three 2.5 cm-diameter coral tissue and skeleton core biopsies were sampled on standard SCUBA dives using a cleaned C.S. Osborne & Company arch punch No. 149 with gloved hands from each of three colonies of both *O. annularis* and *O. faveolata* at 5 m and 12 m water depth (WD), respectively. Each biopsy sample was immediately placed in a Falcon 50 ml polypropylene centrifuge tube at depth, sealed and returned to the surface. Seawater was decanted from each centrifuge tube immediately upon reaching the surface and biopsies were stored in histological fixatives formalin (volumetric mixtures of nine parts ethanol to one part 37% formalin—10% formalin) and Carnoys (volumetric mixtures of three parts ethanol to one part glacial acetic acid). The samples were subsequently shipped and stored at 5 °C in a dark container.

### Optical sectioning fluorescence microscopy and two-photon laser scanning microscopy

The apotome and two-photon microscopy was performed as described in detail before^33,34,58,59^ Briefly, whole non-decalcified biopsies were gently removed from Falcon tubes and placed skeleton-down (polyps facing down) in fresh fixative volumes of the same fixative in which the biopsy was originally preserved in the field. Upon return to Illinois, each biopsy was then placed in a cover-glass bottom chamber (FD5040, WPI, Sarasota, FL). ZEN software was used to control the hardware of the Multiphoton Confocal Zeiss 710 Microscope with Mai Tai Ti–Sapphire laser (Spectra-Physics, Santa Clara, CA), which was utilized for two-photon laser scanning microscopy as described previously^33,34,58,59^. The acquisition parameters are the following: zoom was set at 2, scan speed of 9, line average of 4, 10 µm Z step size, under the 10 × objective, yielding 0.83 µm/pixel resolution.

**Figure 4:**
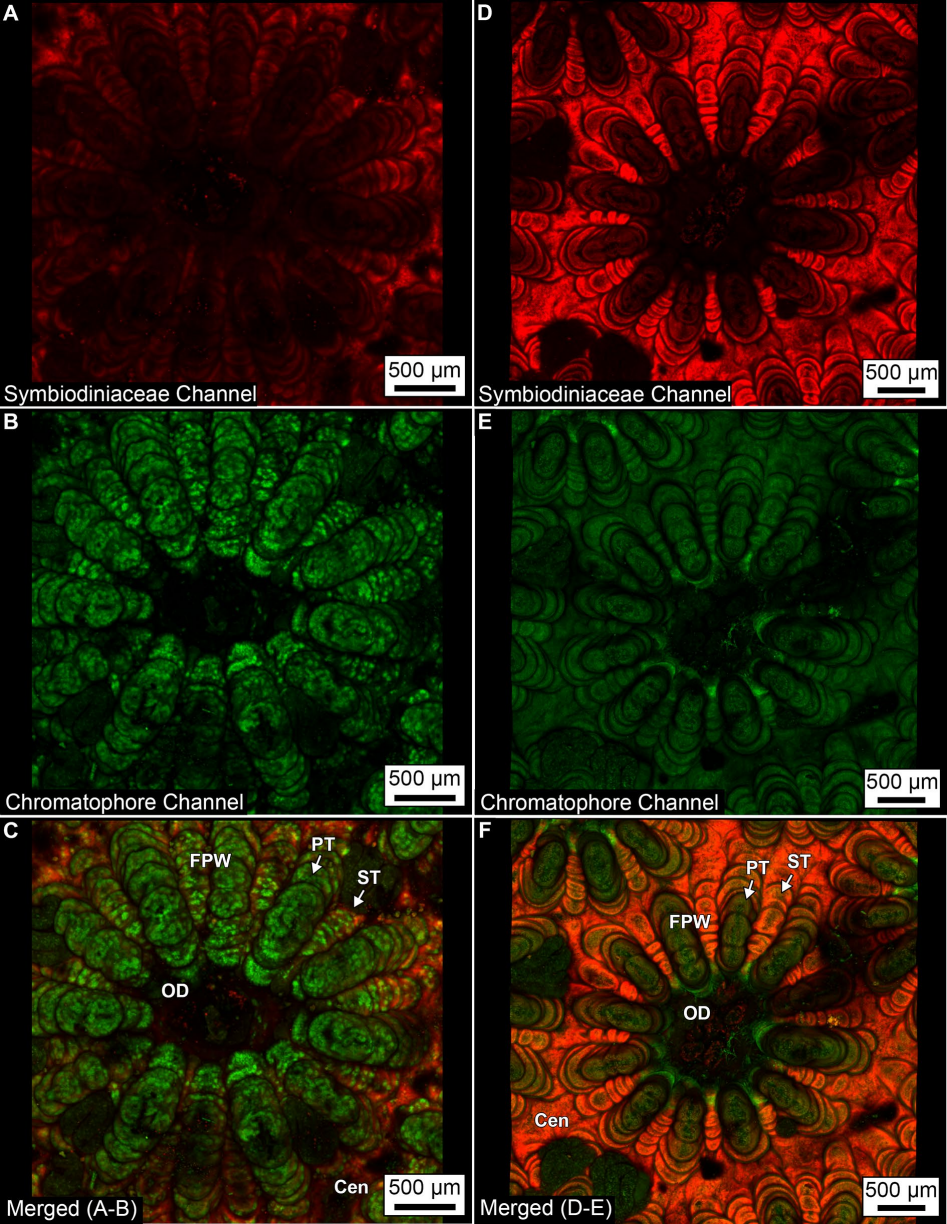
Three-dimensional (3D) two-photon laser scanning microscopy comparing the distribution and abundance of Symbiodiniaceae and chromatopores within and around single polyps of *O. annularis *(A, B, C) and *O. faveolata *(D, E, F). Chromatophore authofluorescene (pseudo-colored green) and Symbiodiniaceae autofluorescence (pseudo-colored red) is reconstructed in 3D from serial optical sections. (**A**) Symbiodiniaceae autofluorescene in *O. annularis*. (**B**) Chromatophore autofluoresence in *O. annularis*. (**C**) Superimposed images of A and B (see also Supplementary Movie 1). (**D**) Symbiodiniaceae autofluorescence in *O. faveolata*. (**E**) Chromatophore autofluorescence in *O. faveolata.* (**F**) Superimposed images of D and E (see also Supplementary Movie 2). Intensities of images in green and red channels were set at the same range (0-149 grey scale in 8 bit) for comparision between species. Lables include: Cen–ceonosarc; FPW–folded polyp wall; PT–primary tentacle; ST–secondary tentacle; and OD–oral disk.

**Figure 5:**
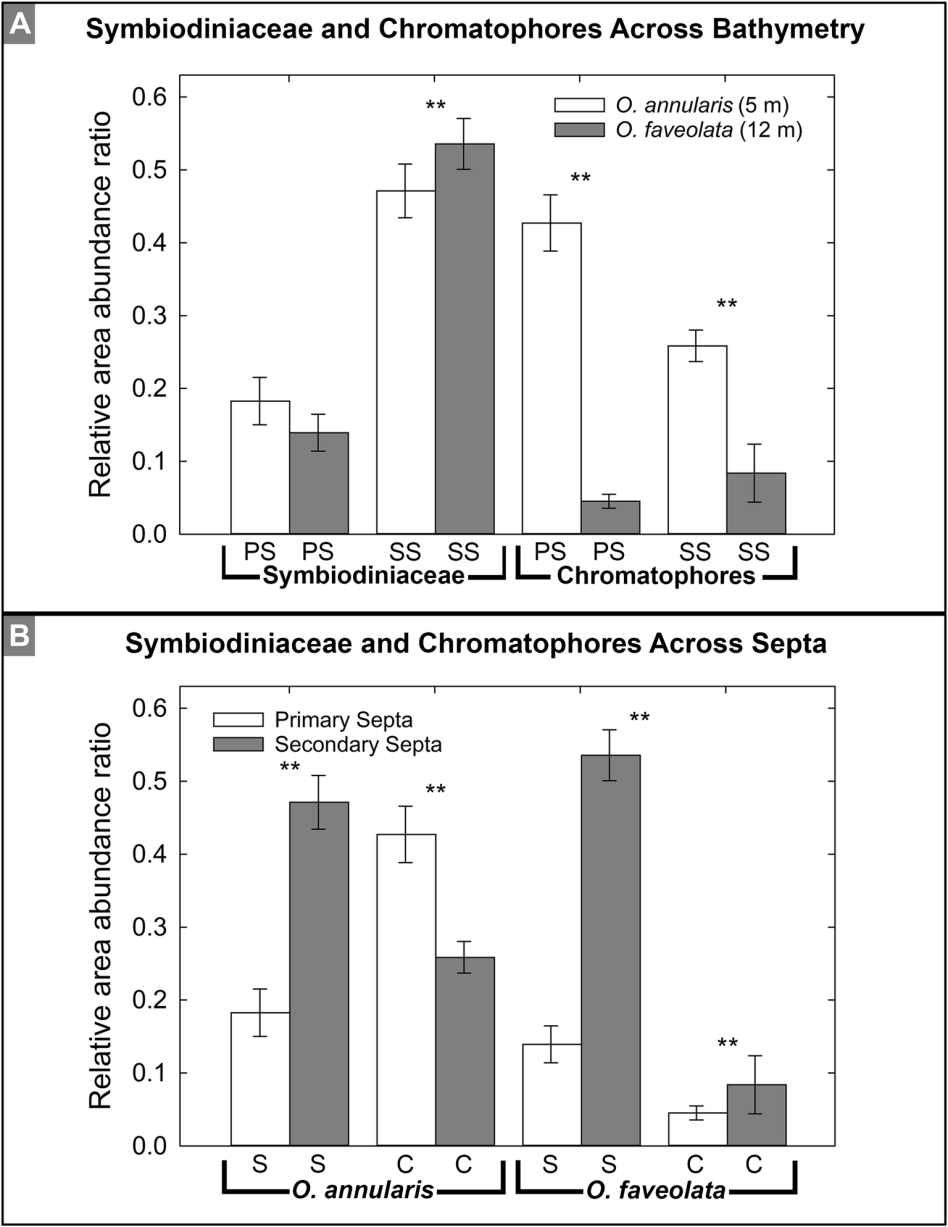
Abundance comparison of Symbiodiniaceae and chromatophores across bathymetry (**A**) and across septa (**B**) in *O. annularis *and *O. faveolata.* Mean values and standard error of normalized relative abundances are presented. Relative abundance shown on the y-axes are a ratio of the area occupied by the target cells divided by the total area in which cells were observed. There was no significant difference in the relative abundance of Symbiodiniaceae in tissues covering the primary septa, but was significant in the tissue overlying the secondary septa. Conversely, relative abundance of chromatophores in the tissues covering both the primary or secondary septa in *O. annularis* was significantly higher that those in *O. faveolata *and all other comparisons between them are significantly different (** = *P* 0.00476) based on the conservative Kruskal-Wallis statistical test. Labels include: PS–primary septa; SS–secondary septa; S–Symbiodiniaceae; and C–chromatopheres. Modified from Miller (2014)^58^.

Autofluorescence imaging of both intrinsic Symbiodiniaceae (chlorophyll *a*) and chromatophores was completed using the two-photon autofluorescence microscopy (TPAF)^33,34,58,59^ The spectral characterization of these components done by both single photon (405 nm and 488 nm) excitation wavelengths and two-photon laser tuned to 780 nm excitation as described previously^34,58^. This was done to analyze how each coral cell components was affected by the differing excitation wavelengths prior to the final selection for analysis. Either internal photomultipliers or spectral photomultipliers were used as detectors. Emission spectra were collected using 405 nm, 488 nm and 780 nm LSM excitation light sources to characterize the emission spectra of individual components such as the oral ectoderm and oral endoderm, as well as the background. The single photon lasers (405 nm and 488 nm) of different wavelengths are limited in the depth of penetration and require a confocal pinhole in order to create an optical section. The two-photon laser provides an inherent confocality exhibiting no out of focus light or loss of excitation emission when penetrating deep through the tissue samples^34,58,60–63^. These lasers were tested on coral Symbiodiniaceae and chromatophores to determine the optimal strategy for characterizing the associated autofluorescence in future studies. Both cell types were identified in *O. annularis* and *O. faveolata* using identical excitation wavelengths as well as collected at the polyp tissue wall (Figs. 4, 5; SI Figs. S1–S4, S11 and SI Movies 1–64). Signals were spectrally unmixed using the built in unmixing algorithm to recover the spectral profiles of components. On the other hand, the green and red band pass filters were used in front of two PMT detectors to collect simultaneously the autofluorescence from the chromatophores (pseudo-colored green) or from the chlorophyll *a* from the Symbiodiniaceae (pseudo-colored red) for 3D reconstruction and quantifications as described in detail previously^33,34,58,59^. Images of 3–4 polyps at two different magnifications (digital zoom) using a 10X (0.3 NA) objective were obtained as described previously^33,34,58,59^. The coral polyp shape and height varies between samples (usually 1–2 mm) and the imaging depth is also limited by the imaging objective’s working distance. Two PMT detectors were used simultaneously to collect the data from two components *i.e*., one from 500 to 550 nm (green-chromatophore autofluorescence) and the other 650–720 nm (red-chlorophyll Symbiodiniaceae fluorescence). The tile scan mode collected ~ 25–100 (5 × 5 or 10 × 10) tile images per focal plane in x–y axis and 50–100 tile images through the z axis at 10 or 20 µm interval, totaling around 5000–10,000 images/coral polyp. The images were subsequently rendered in 3D in Imaris program as described previously^33,34,58,59^. These images of three-dimensional distribution of the fluorescent components excited by 780 nm wavelength of *O. annularis,* found in 5 m WD, and *O. faveolata,* found in 12 m WD. These samples were collected in January 2010 and show the plastic, light-optimizing strategy of the host coral (green, fluorescent pigments in host cell chromatophores found in the oral ectoderm) and the underlying Symbiodiniaceae (red, Symbiodiniaceae auto-fluorescence found in the oral endoderm and calicoblastic epithelium) in response to only 7 m WD difference. This drastic change in light-harvesting or photo-protective strategies is expressed over a very small change in water depth (0-10 m water depth difference in inhabited ecological niches), making these corals able to regulate the spatial and temporal distribution of Symbiodiniaceae and chromatophores and therefore permit the coral holobiont to successfully acclimatize.

### Quantification of Symbiodiniaceae and chromatophore abundances

The individual channel intensity of raw data for Symbiodiniaceae and chromatophores is thresholded using the Imaris 3D-Spot Isosurface rendering algorithm and volumes are developed in the same program (Bitplane Inc., Zurich, Switzerland)^33,34,58,64^. Individually, each tentacle tissue overlying the primary and secondary septa were traced using contour tracing algorithm in the same program to precisely quantify the Symbiodiniaceae and chromatophore cells based on data from high magnification images of multiple polyps (Supplementary Fig. S11). The cell count of Symbiodiniaceae or chromatophores within each tissue overlying a primary or secondary septa was divided by the total volume of that cell type found within entire polyp to statistically normalize the data for overall comparison between varying sized polyps. The images are displayed as both raw data with maximum intensity projection as well as rendered data in 3D-Spot-Isosurface algorithm, from which the volume quantification data is obtained (Supplementary Fig. S4).

### Symbiodiniaceae autofluorescence, biomolecule immunolocalization and quantification

*O. faveolata* tissue-skeleton biopsies, collected in March and May of 2008, were decalcified in 10 mM EDTA solution for 12 weeks until the entire calcium carbonate skeleton was slowly and gently dissolved. The de-mineralized tissue biopsies were prepared for the thin section preparation as described previously^33,34^. The tissue was briefly dehydrated in a series of increasing concentrations of ethanol in PBS, then exchanged in 100% ethanol three times, infiltrated and embedded in paraffin wax. Following this, 6–8 µm thick sections were made in a microtome that were expanded and adhered to a glass slide. The sections on the slides were dewaxed using 100% xylene three times and rehydrated in increasing water to ethanol series. Then they were blocked with IT signal-Fx blocking reagent cocktail (Invitrogen, Carlsbad, CA) with two primary monoclonal antibodies. The first was made in mouse against calmodulin (MA3-918 calmodulin monoclonal antibody 6D4, Invitrogen, Carlsbad, CA) and the second was a custom-made affinity purified polyclonal primary antibody against Anti-STPCA, using a peptide sequence of ‘RDP EGP DTW KHH YKDC’ specific to STPCA and raised in Rabbit from Genscript Biotech Corp., NJ. This is a carbonic anhydrase specific conserved sequence identified by Moya et al.^65^, from a Scleractinian reef building coral *Stylophora pistillata*. In addition, samples were incubated with wheat germ agglutinin (WGA) to label the sialic acid residues (N-acetyl glucosamine) in the coral mucus. The primary antibodies were probed with appropriate secondary antibodies either anti-rabbit Alexa350 (for STPCA) or anti-mouse Alexa546 (for calmodulin; Invitrogen, Carlsbad, CA) together with WGA Alexa488 fluorescence conjugate.

In preparation for microscopy, the sections were mounted in Prolong Gold, an antifade mounting medium to prevent the photobleaching, and imaged in a widefield microscope with an Apotome, which is an optical sectioning tool which rejects out of focus light from widefield fluorescencel^33,66^. The auto-fluorescence of Symbiodiniaceae chlorophyll fluorescence was recorded in Alexa 647/Cy5 channel and all other target fluorescence were recorded with appropriate filters in a wide-field fluorescence microscope using metal halide mercury lamp as a light source (Axiovert M200, Zeiss, Germany) and the Axiovision software (Zeiss, Germany). The imaging was performed using a Zeiss Axiocam Apotome optical sectioning fluorescence microscope^66^ using a high resolution monochrome camera to capture four channel images. The standard filter sets were used to capture raw black and white images using fluorescence microscopy (FM) in the following specific channels: blue (DAPI filter with an excitation (Ex) of 300–400 nm and emission (Em) of 440–500 nm), green (FITC filter; Ex: 460–500 nm; Em: 510–560 nm), orange (Rhodamine filter; Ex: 540–550 nm; Em: 580–640 nm) and red (Cy5 filter; Ex: 630–650 nm; Em: 660–700 nm).

**Figure 6:**
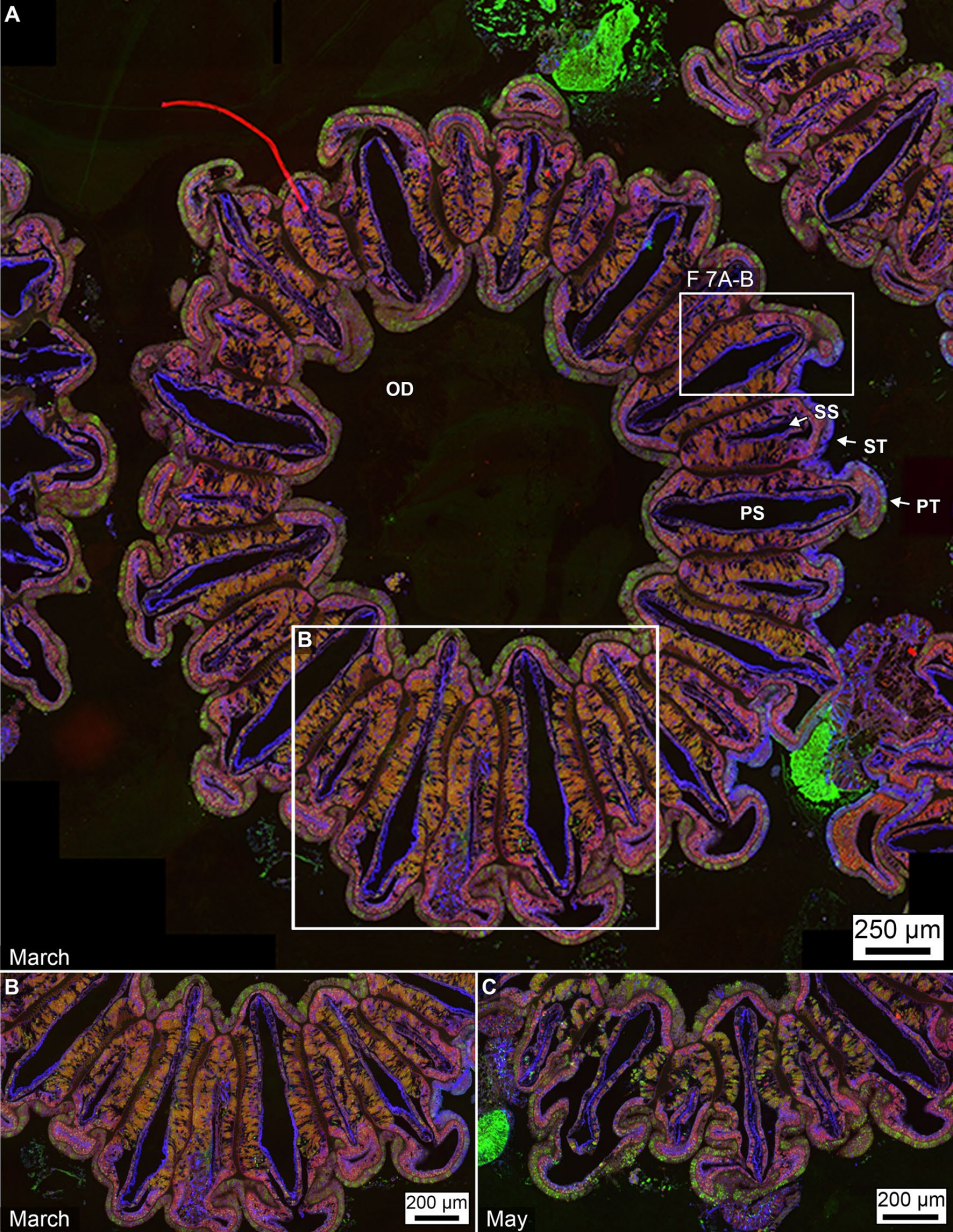
Immunohistochemical fluorescence microscopy of the distributions and abundance of Symbiodiniaceae, chromatophores carbonic anhydrase, calmodulin and mucus within tagged polyp tissues of *O. faveolata*. (**A**) Horizontal cross-section of decalcified polyp tissues of *O. faveolata* collected in March 2008 of superimposed emissions from mucus (blue fluorescence), carbonic anhydrase (green fluorescence), and calmodulin (red fluorescence). (**B**) Enlargement of the box shown in A. (**C**) Horizontal cross-section of a decalcified polyp of *O. faveolata* collected in May 2008 of superimposed mucus (blue fluorescence), carbonic anhydrase (green fluorescence), and calmodulin (red fluorescence). Enlargement from the box shown within a full polyp shown in Supplementary Fig. S7. Labels include: PS—primary septum; SS—secondary septum; PT—primary tentacle; ST—secondary tentacle; and OD—oral disk.

**Figure 7:**
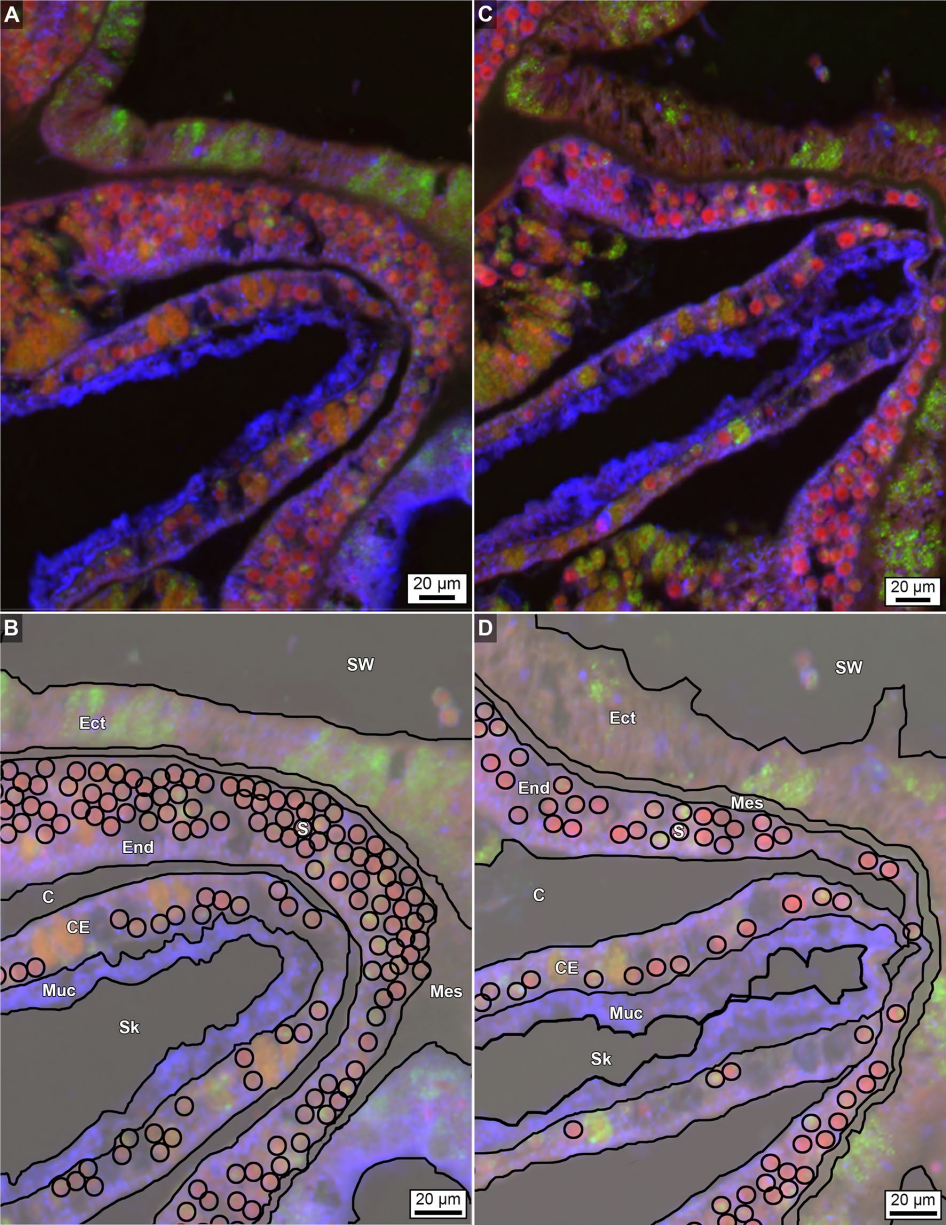
Enlargement of immunohistochemical fluorescence microscopy of the distributions and abundance of carbonic anhydrase, calmodulin and mucus within tagged polyp tissues of *O. faveolata*. (**A**) Original enlarged image (from box 7A-B shown in Fig. 6A) of a horizontal cross-section of decalcified polyp tissues of *O. faveolata* collected in March 2008 exhibiting superimposed emissions from mucus (blue fluorescence), carbonic anhydrase (green fluorescence), and calmodulin (red fluorescence). (**B**) Line tracing of A indicating individual Symbiodiniaceae. (**C**) A second enlargement (from area shown in Supplementary Figs. S5, S6) image of a horizontal cross-section of decalcified polyp tissues of *O. faveolata* collected in May 2008 exhibiting superimposed emissions from mucus (blue fluorescence), carbonic anhydrase (green fluorescence), and calmodulin (red fluorescence). (**D**) Line tracing of C indicating individual Symbiodiniaceae are found in both the oral endoderm and the calicoblastic epithelium. Labels include: End—endoderm; Mes—mesoglea; Ect—ectoderm; Muc—mucus; PS—primary septum; SW—seawater at the time the polyps were collected; and Sk—skeleton. Tissue structure terminology follows nomenclature in Allemand et al^54^ and Peters^55^.

**Figure 8:**
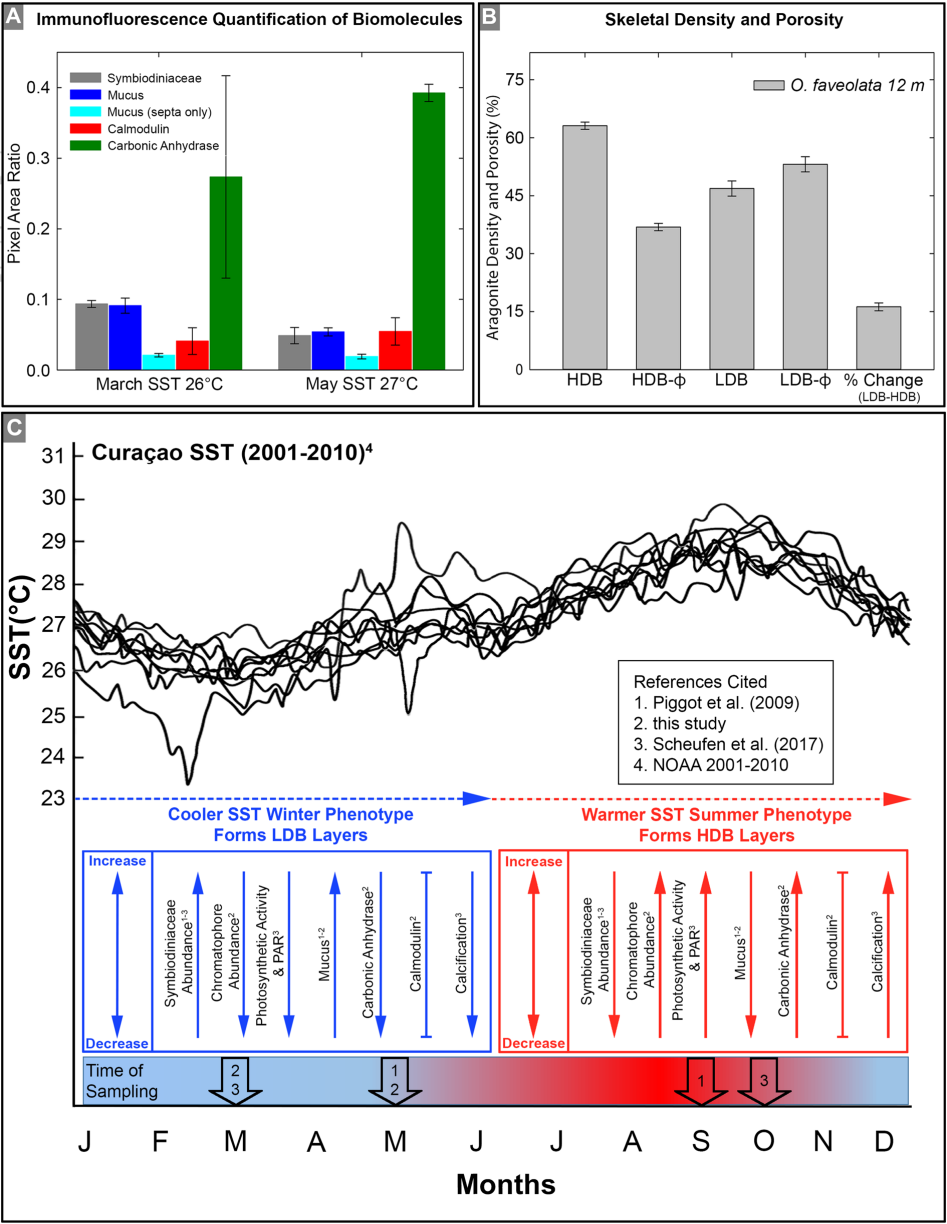
Quantification of Symbiodiniaceae and biomolecule tissue abundances, as well as coral skeletal density banding (CSDB), in biopsies of *O. annularis* and *O. faveolata*. (**A**) Quantification of Symbiodiniaceae and biomolecule tissue abundances in decalcified whole polyps of *O. faveolata*. Analyses were completed at a tissue depth of 800 µm below the uppermost polyp surfaces. All abundance measurements were normalized to the total tissue cross-sectional area of each polyp. Standard deviation error bars were calculated from measurements collected in triplicate. (**B**) Quantification of the percent density and porosity change from individual HDB and LDB layers within *O. faveolata*. (**C**) Summary of annual changes observed during seasonal acclimatization in *O. annularis* during seasonal transitions from cooler SST winter phenotype to the warmer SST summer phenotypes. Significant variations in annual SST on Curaçao (NOAA 2001–2010) provide a valuable context for evaluating changes in the tissue abundance of Symbiodiniaceae cells and biomolecules in *O. annularis*. In addition, other key components were included from the combined results of previous studies^30,33^.

Some spectral emission overlap, called “spectral bleed-through”, was observed when using different excitation wavelengths of light. This effect may reach ~ 10–20% between autofluorescent channels where the target is not specifically tagged by an antibody or fluorophore and one of those autofluorescent components is very bright, such as Symbiodiniaceae and chromatophore channels (Figs. 4, 6, 7, 8; Supplementary Figs. S5–S10). To minimize this effect, image overlay was done only using three channels for clarity (carbonic anhydrase, calmodulin and mucus) and merged for quantitative analysis. This included localization of individual components (blue, green, and red) and the autofluorescence channel for Symbiodiniaceae was not used for this purpose (Figs. 6, 7; Supplementary Figs. S5–S10). In these images, each component is consistently pseudo-colored. The equal overlap of green and red channels exhibit yellow-orange light, green and blue overlap exhibits cyan, and red and blue overlap produce magenta. The combination of all three creates white light. If red and blue channels are merged and an image contains a stronger emission of red compared to blue, then a pink light is produced at that given location. Conversely, if the blue light channel emission is higher in intensity relative to the red, then a deep purple color is seen in the image. The image analyses in the present study, which were modified from Piggot et al.^33^, permit an objective selection of immuno-labeled components using a threshold of fluorescent intensity (Figs. 6, 7; Supplementary Figs. S5–S10). In the newly developed technique, the area of all pixels exhibiting the range of selected intensities was totaled to quantify the total area occupied by that particular tissue cell constituent (Fig. 5). This methodology has resulted in the first spatial and temporal quantitative documentation of the distribution and abundance of coral Symbiodiniaceae, chromatophores, carbonic anhydrase, calmodulin protein and mucus in Orbicella corals.

### Statistical evaluation and analyses

Statistical analyses were performed as described previously^40,67–69^. Briefly, three different kinds of statistical analyses were applied to identify significance and correlations between the chromatophore and Symbiodiniaceae relative abundance measurements. In the first step, the data set included the variables of water depth (WD), polyp lobe type, coral tissue cell type and relative abundance (RA). In the second step, the data set included water depth (at either 5 m or 12 m), tissue cell type, and RA. Lastly, these two data sets were combined into one data set that included the variables water depth, tissue cell type and RA. Though water depth was a continuous variable, it was treated as a factor because there were only two unique values of 5 m WD and 12 m WD. This was done because it is not possible to validate a linear relationship between RA and water depth with two values. Although the output from this original parametric ANOVA analysis cannot be formally accepted due to the non-parametric nature of the dataset: the p-values for all the interaction terms were extremely small (ranging from 5e^-4^ to 2.6e^-9^), indicating that the important factor was the combination of these three independent variables (such as each combination of water depth, polyp lobe type, and tissue cell type had its own mean RA). Therefore, it is reasonable to consider eight groups (two species + two water depths + two polyp lobe types + two tissue cell types = 8 groups). This makes the eight group data set equivalent to a one-way (non-parametric) ANOVA analysis.

Pairwise differences were evaluated using the Kruskal–Wallis as described previously^34,39,58,67,69^. Nonparametric rank sum test. Each pair of combinations were tested to identify significant differences. The mean estimates and the significant pairwise differences for each combination or comparison (i.e., Symbiodiniaceae on primary septa at 5 m WD versus Symbiodiniaceae on primary septa at 12 m WD). Each group was analyzed for statistically significant differences in relative abundance using this method, resulting in 21 tests. To account for the multiple tests, the significance level was adjusted accordingly. Type I error was the probability of rejecting the null hypothesis, when in fact the null hypothesis was true. Hence, to make appropriate adjustments, the significance level was divided by the number of tests performed. While this approach is extremely conservative, it guaranteed to control overall type I error. Two datasets were considered to have statistically significant different means if the p-value for their pairwise Kruskal–Wallis test was less than 0.1/21 = 0.00476. This adjustment is referred to as the Bonferroni correction^34,39,58,67^. Statistically significant changes in relative abundance were found in all tested eight groups, except for the pairwise comparison between the Symbiodiniaceae found in the primary septa of the 5 m and 12 m WD experiments.

## Results and discussion

### Symbiodiniaceae and chromatophore tissue distribution and abundance across bathymetry

Symbiodiniaceae in *O. annularis* and *O. faveolata* exhibited a fluorescence emission peak from chlorophyll *a* at ~ 670 nm when excited with both 405 nm (blue light) and 780 nm (two-photon near infrared light) wavelengths (Supplementary Figs. S1–S4). In corals, chlorophyll *a* is accompanied by chlorophyll *c*, and hence there is no discrimination made between them^70^. In both *O. annularis* and *O. faveolata*, Symbiodiniaceae cells were observed in the oral endoderm and are distributed throughout the entire polyp from the oral disk to the coenosarc, with the only exceptions being the most distal tips of the primary and secondary tentacles (Fig. 4; Supplementary Figs. S1–S4; Movies 1, 2). In addition, Symbiodiniaceae were detected within the calicoblastic epithelium in both *Orbicella* species (Fig. 4; Supplementary Figs. S1–S2; Movies 1, 2), which has not been previously observed in other coral species^42,54^. Qualitative two-photon laser scanning microscopy 3D projections of whole polyps indicate that the resulting autofluorescence emission intensity is significantly higher by 7–8% in the deeper water *O. faveolata* tissues than in the shallower water *O. annularis* tissues^34^ (Fig. 4; Supplementary Figs. S1, S2). Because the Symbiodiniaceae are in the oral endoderm in *O. annularis* and there is a significantly higher abundance of chromatophores in the oral ectoderm, 3D projections of Symbiodiniaceae emissions in *O. annularis* are attenuated and artificially appear darker (Fig. 4; Supplementary Figs. S1, S2). However, the significantly lower abundance of chromatophores in *O. faveolata* do not attenuate the Symbiodiniaceae emissions (Fig. 4; Supplementary Fig. S1). As a result, the Symbiodiniaceae emissions of the entire polyp in *O. annularis* appear weaker with respect to the Symbiodiniaceae emissions observed in *O. faveolata* (Fig. 4; Supplementary Figs. S1, S2). The highest abundance of Symbiodiniaceae in *O. annularis* occurs in the coenosarc (Fig. 4A,C), while the highest abundance of Symbiodiniaceae in *O. faveolata* is in the folded polyp wall, coenosarc and tentacles overlying the secondary septa (Figs. 4D,F, 5). Conversely, the lowest abundance of Symbiodiniaceae in *O. annularis* tissues is in the folded polyp wall tissues and tentacles overlying both the primary and secondary septa (Figs. 4, 5; Supplementary Fig. S1), while the lowest abundance of Symbiodiniaceae in *O. faveolata* is in the tentacles overlying the primary septa (Figs. 4 and 5; Supplementary Fig. S1; Movies 1, 2).

Chromatophores in both *O. annularis* and *O. faveolata* occur in the oral ectoderm throughout the entire polyp from the oral disk to the coenosarc and directly overlay Symbiodiniaceae that are within the oral endoderm (Fig. 4; Supplementary Figs. S1, S2; Movies 1, 2). No chromatophores were observed in the present study within the calicoblastic epithelium, which is consistent with previous reports in other corals^54,71^. The highest abundance of chromatophores in *O. annularis* is in the tissue overlying primary and secondary septa, while in *O. faveolata* the highest abundance is in tentacles overlying the primary septa as well as the tentacle tips overlying the secondary septa (Figs. 4, 5). Conversely, while the lowest abundance of chromatophores in *O. annularis* is in the coenosarc, the lowest abundance in *O. faveolata* is in the folded polyp wall tissues and tentacles overlying the secondary septa (Figs. 4; Supplementary Figs. S1, S2). This inverse covariation of increasing chromatophore abundance accompanied by decreasing Symbiodiniaceae occurs in both *O. annularis* and *O. faveolata* (Figs. 4, 5; Supplementary Figs. S1, S2; Movies 1, 2). Quantitative comparison of the 3D two-photon imaging of *O. annularis* at 5 m WD with *O. faveolata* at 12 m WD (Fig. 5) indicate that there were no statistically significant differences in the abundance of Symbiodiniaceae in the tissues covering the primary and secondary septa (described in the statistical evaluation and analysis section; Fig. 5A). In addition, the abundance of chromatophores in *O. annularis* is significantly higher in the tissues overlying the primary and secondary septa than those in *O. faveolata* (Fig. 5). Furthermore, the abundance of Symbiodiniaceae in whole polyps is 25% higher in *O. faveolata* tissues than in *O. annularis* tissues^34^ (Fig. 4; Supplementary Figs. S1, S4, S11). Within both species, the tissues overlying the secondary septa generally exhibit a significantly higher abundance of Symbiodiniaceae than the tissues covering the primary septa (Fig. 5). Chromatophore abundance in whole polyps of *O. annularis* is 133% higher than in *O. faveolata*, while chromatophores are preferentially located in the tissues covering the primary septa in both species (Fig. 5B). Conversely, *O. faveolata* exhibits no significant difference from *O. annularis* in the abundance of chromatophores in the tissues covering the primary and secondary septa (Fig. 5B; Supplementary Figs. S4, S11; Movies 3–6). Furthermore, within *O. annularis* the 3D two-photon microscopy volume measurement of chromatophores is 38% greater than the volume of Symbiodiniaceae, while in contrast within *O. faveolata* the volume of chromatophores is 56% lower than the volume of Symbiodiniaceae (Supplementary Figs. S4, S11; Movies 3–6). Tracking these autofluorescence emissions within the intact original 3D tissue structure indicates that Symbiodiniaceae primarily occur in the oral endoderm and fewer are present in the calcioblastic epithelium, while the chromatophore emissions are exclusively from the oral ectoderm (Supplementary Figs. S4, S11; Movies 3–6).

### Symbiodiniaceae and biomolecule distribution and abundance correlated with water depth, seasonal SST and CSDB

Primary and secondary antibodies targeting carbonic anhydrase, calmodulin and mucus were compared between samples collected from the same colony of *O. faveolata* at 12 m WD in March 2008 (SST = 26.0 °C ± 0.03 °C at the end of the winter season) and May 2008 (SST = 27.0 °C ± 0.03 °C in the middle of the spring; Figs. 6, 7 and 8; Supplementary Figs. S5–S10. This allowed comparison of their distribution and abundance within individual decalcified *O. faveolata* whole polyp tissue histology sections (Figs. 6, 7, 8). In addition, this permitted mapping within individual whole *O. faveolata* polyps (Fig. 7A), in March 2008 (Fig. 7B, 8A,C, SI Figs. S5–S7) and May 2008 (Fig. 7B,D, SI Figs. S8–S10), the distribution of individual biomolecules and their respective segregation or overlap within the intact tissue structure (oral ectoderm and oral endoderm). In contrast to previous models for general coral tissue structure^54^, these analyses indicate that *O. faveolata* does not possess an aboral endoderm (Fig. 7). Similarly, Symbiodiniaceae in *O. faveolata* were observed in both the oral endoderm and calicoblastic epithelium (Fig. 7). Polyps of *O. faveolata* collected in March 2008 exhibited higher abundances of Symbiodiniaceae and mucus, lower carbonic anhydrase and no significant differences in calmodulin compared to *O. faveolata* polyps collected in May 2008 (Figs. 6, 7, 8). These shifts in biomolecule abundance are graphically depicted by image tracings (Fig. 7B, D) and are generally consistent with those presented in Piggot et al.^33^ with respect to mucocytes in the oral ectoderm. However, quantification of images in the present study from the calicoblastic epithelium shows a decrease in the abundance of mucus and Symbiodiniaceae going from polyps collected in cooler March seawater to polyps collected in warmer May seawater (Fig. 8). These trends also correlate with increased abundance of carbonic anhydrase, while the calcium-binding protein calmodulin remains relatively constant (Fig. 8).

### Implications for water depth and seasonal acclimatization of the coral holobiont

A comprehensive understanding of the underlying physiological and biochemical processes that govern seasonal acclimatization of the *Orbicella* holobiont is beyond the scope of the present study. However, as a step toward this eventual goal, multiple working hypotheses are presented in the following that combine the spatial and temporal distribution of Symbiodiniaceae cells and biomolecules documented in the present work (Figs. 4, 5, 6, 7, 8A) together with reports from previous studies (e.g.,^30,33^; Fig. 8C). Tissues in both *O. annularis* and *O. faveolata* contain seasonally variable, yet overall high abundances of Symbiodiniaceae cells (Figs. 4, 5, 6, 7, 8A;^30,33^). This is manifested as a cooler SST winter phenotype that transforms into a warmer SST summer phenotype (Fig. 8C). This annual process of seasonal acclimatization of the coral holobiont includes: (1) a decrease in the tissue abundance of Symbiodiniaceae cells (Figs. 4, 5, 6, 7, 8A;^30,33^); (2) an increase in Symbiodiniaceae photosynthetic activity^30^; (3) an increase in the tissue abundance of chromatophore cells (Figs. 4, 5, 6, 7, 8A; SI Figs. S5–S10); (4) a decrease in mucus production (Fig. 8A;^33^); (5) an increase in carbonic anhydrase production (Fig. 8A); (6) no appreciable change in calmodulin (Fig. 8A); and (7) an increase in calcification rate^30^).

Recent reports have demonstrated that lower Symbiodiniaceae abundances in the *Orbicella* summer phenotype are accompanied by higher rates of photosynthesis, both per symbiont and per total coral tissue area (Fig. 8C;^30^). As a result, symbiont photosynthetic activity increases in the summer phenotype despite significant declines in symbiont cell abundance (Fig. 8C;^30^), perhaps acting as a compensation mechanism for the decrease in the abundance of Symbiodiniaceae. As a result, trophic transitions away from Symbiodiniaceae-based autotrophy toward coral heterotrophic feeding is not obligatory during higher SST^30^. This trophic plasticity in *O. annularis* and *O. faveolata*^72^ may therefore represent a fallback strategy for nutrient assimilation by the host coral whenever Symbiodiniaceae autotrophy is unavailable or insufficient. The inherent flexibility of trophic plasticity may be especially advantageous during the highly variable year to year changes in the timing, rate and magnitude of SST (Fig. 8C) during the course of the life and survival of corals through geological time.

Covariations observed between the distribution and abundance of Symbiodiniaceae and chromatophores imply that chromatophores may serve multiple functional purposes (Figs. 4; Supplementary Figs. S1, S4 Movies 1–6). On the one hand, *O. annularis* and *O. faveolata* strategically position and increase the abundance of chromatophores in the oral ectoderm in response to changes in both SST and WD (Fig. 4, SI Figs. S1–S4, and Movies 1–6). This implies that the green fluorescent proteins serve to photo-protect Symbiodiniaceae in the underlying oral endoderm^32^. On the other hand, there is also an inverse relationship between Symbiodiniaceae and chromatophore tissue abundance (Figs. 4; Supplementary Figs. S1, S2; Movies 1, 2). This is exemplified by Symbiodiniaceae abundance being greatest in the coenosarc where chromatophore abundance is at its lowest (Fig. 4). Although no direct evidence was collected in the present study to further evaluate this possibility, these observations imply that chromatophores may serve other functional purposes for the coral holobiont in addition to photo-protection for the Symbiodiniaceae.

Regarding the process of photoprotection, similar bulk coral polyp tissue Symbiodiniaceae and chromatophore emissions at varying depths has previously been reported from bulk tissue analyses of the reef building corals *Acropora nobilis*, *Porites cylindrica* and *Montipora digitata* on the Great Barrier Reef^32^. This effect is further accentuated in the shallow water *O. annularis*, where the abundance of chromatophores is significantly increased in response to increased PAR and ultra-violet radiation (UV)^4,73^. Furthermore, the distribution and abundance of Symbiodiniaceae, which is equivalent in both *O. annularis* and *O. faveolata*, is therefore likely to be strongly influenced in the environment by PAR^18^. Small changes in PAR occur across the small microscale topography of the polyp and surrounding tissues. For instance, while the highest elevation tentacle tissues at the top of the polyp are exposed to the highest PAR, the highest abundance concentration of Symbiodiniaceae occur in lower tissues to be protected from reactive oxygen species that cause photo-inhibition^1,74–76^. Qualitative and quantitative analyses of Symbiodiniaceae distribution and abundance in *O. annularis* and *O. faveolata* in the present study have identified (Figs. 4, 5): (1) an ~ 50% decrease of Symbiodiniaceae in the uppermost folds of the tentacle tissues covering the primary septa; (2) a decrease in Symbiodiniaceae in the uppermost folds of the tentacle tissues covering the secondary septa; (3) an increase in Symbiodiniaceae abundance in the coenosarc. The exact mechanism by which specific wavelengths of light interact with the green fluorescent proteins in chromatophores, and how the lethal radiation is absorbed and not transmitted down to the Symbiodiniaceae layers is unknown. However, stereochemical changes in the configuration of amino acid residues have been proposed as a potential mechanism^77,78^. Taken together, this evidence indicates that Symbiodiniaceae photosynthesis and chromatophore photo-protection from UV light is most pronounced in shallow water *O. annularis*. In addition, the coenosarc and the primary and secondary tentacle tissues are the sites of the highest abundance and photosynthetic activity of Symbiodiniaceae in both *O. annularis* and *O. faveolata* (Fig. 4).

Complex biotic and abiotic factors that combine to influence seasonal acclimatization also influence the crystalline structure and stratigraphy of coral skeletal growth^79–82^. However, precisely how coral skeletal density banding (CSDB) sequences, which are composed of macro- and micro-scale high density band (HDB) and low density band (LDB) layering, remains controversial (Fig. 8, SI Figure; e.g.,^7,83^. The successful functioning and survival of the coral holobiont and its impact on resulting CSDB depends on the simultaneous influence of complex intertwined environmental (e.g., SST, water depth, sedimentation, currents, nutrient and oxygen availability, and seafloor diagenesis) and biological factors (e.g., photo-pigmentation, electron transfer rates, CO_2_ availability, host coral biomass^84–87^). Especially influential for CSDB formation during seasonal acclimatization from winter to summer *Orbicella* phenotypes is how calcium anhydrase (CA) and calmodulin activity changes with SST, photosynthetically active radiation (PAR), rate of Symbiodiniaceae photosynthesis, and CO_2_ availability^85,88^. However, because the total rate of Symbiodiniaceae photosynthesis increases as tissue cell abundance decreases when transitioning from the winter to summer phenotype (Fig. 8C;^30^), it is likely that a high aragonite saturation state is maintained in the calcifying space (mucus) between the calicoblastic epithelium throughout the year^54,89–92^. As a result, other yet to be identified processes, such as protein catalysis^93^, may also play a role.

Throughout the seasonal acclimatization process of the coral holobiont, mutualism between Symbiodiniaceae and its *Orbicella* host depends on the exchange and fixation of carbon^94,95^. The coral provides its respired CO_2_ as well as dissolved inorganic carbon (DIC) from seawater, which is then fixed during Symbiodiniaceae photosynthesis and translocated back to the coral as organic carbon nutrients. Remarkably, Symbiodiniaceae photosynthesis is capable of fulfilling as much as 95% of the metabolic requirements of the coral host when at maximum activity^96^. In order for *Orbicella* to continue to precipitate skeleton throughout the seasonal acclimatization process, the coral carbonic anhydrase (CA) converts HCO_3_^-^ into CO_2_ and thus sustains Symbiodiniaceae photosynthesis^95,97,98^. The CO_2_ concentrating mechanisms of CA has a greater potential for fixing photosynthetic carbon at higher SST^94,95^, as reflected by the observed increase in CA abundance in the summer phenotype (Fig. 8A,C). This will in turn shift the balance of carbon translocation between Symbiodiniaceae and the host coral and impact skeletal calcification^99^. These carbon translocation processes, production of fatty acids and other lipids by the host coral during feeding and by Symbiodiniaceae under variable PAR conditions, will also play a role in the formation of HDB and LDB layers (Fig. 8B;^100^).

A custom developed antibody, which is made against *Stylophora pistillata* and universally conserved in scleractinian corals^65,101–104^, provided the required immunohistochemical specificity for *O. faveolata* CA abundance in the present study (Figs. 6, 7, 8; Supplementary Figs. S5–S10). The observed change in CA is not statistically significant between March and May 2008, which is presumably due to the small 1 °C change in SST (Fig. 8). However, the mean intensities of CA from March to May imply a relatively increasing trend with increasing SST (Figs. 6, 8A), which is consistent with previous studies^94,95^. Quantification via micro CT imaging indicates that HDB layers are 16% more dense and less porous than the immediately above and below corresponding LDB skeletal layers (Fig. 8B). Furthermore, this correlates closely with changes in the abundance of Symbiodiniaceae, mucus seasonal SST oscillations (Fig. 8C).

Calmodulin, a membrane-bound calcium binding protein that is utilized as an indicator for calcium availability, remained relatively unchanged in *O. faveolata* during both March and May 2008 (Fig. 8A,C, SI Figs. S5–S10). These results are consistent with previous studies that indicate calcium availability is a non-rate-limiting step in coral skeletogenesis and that it is actively transported across the calicoblastic epithelium through the light-activated Ca^2+^-ATPase pump^54,85,102^. Mucus, another component related to seasonal acclimatization, is composed of sialic-acid residues, polysaccharides (sugars) and secreted by mucocytes in the oral ectoderm, the oral endoderm and the calcifying space (Figs. 6, 7, SI Figs. S5–S10). Previous studies have primarily focused on mucus produced in the oral ectoderm and oral endoderm that is used by the coral to form the protective coral surface microlayer^19,105,106^. In the present study, all mucus within the oral endoderm, oral ectoderm, calicoblastic epithelium and the calcifying space was labelled and quantified (Figs. 6, 7, 8; Supplementary Figures S5–S10) using wheat germ agglutinin (WGA) targeted against sialic acid residues, which are primarily N-acetylglucosamine^19,54,71,93,105–111^. Mucus abundance increased from March to May 2008 and closely correlates with Symbiodiniaceae abundance (Fig. 8). It has been proposed that the secreted mucus closely associated with Symbiodiniaceae could be a by-product of photosynthesis in the oral endoderm and might serve either as energy storage or play a pivotal role in host-recognition of the endosymbiont^107,111,112^. In addition, mucus within the calcification space (Figs. 6, 7, 8), in coordination with mucocytes in the oral ectoderm and the oral endoderm, act in tandem as a targeted conduit for direct seawater influence on formation of encapsulated amorphous calcium carbonate (ACC)^113^.

In conclusion, shallower 5 m WD *O. annularis* biopsies exhibited a decrease in the abundance of Symbiodiniaceae cells and an increase the abundance of chromatophores. Conversely, deeper *O. faveolata* 12 m WD exhibited inverse relationships of increasing Symbiodiniaceae and decreasing chromatophores. Furthermore, inverse covariations observed between the distribution and abundance of Symbiodiniaceae and chromatophores imply that chromatophores may serve multiple functional purposes in addition to photo-protection. Seasonal acclimatization of the *O. faveolata* holobiont observed in the present study and previous reports, suggests that biomolecules (chromatophores, calmodulin, carbonic anhydrase and mucus) are differentially produced during acclimatization from cooler to warmer SST. Decreased Symbiodiniaceae abundance in summer SST phenotypes are accompanied by higher rates of photosynthesis, which compensates for the decrease in Symbiodiniaceae. This implies that trophic plasticity toward coral heterotrophic feeding, while not required during higher SST, remains a fallback metabolism available throughout the year when environmental stress reduces Symbiodiniaceae autotrophic capacity. The transition to warmer SST was also accompanied by decreased mucus production, decreased Symbiodiniaceae abundance and increased photosynthetic activity that combines to enhance calcification. These complex interacting processes that facilitate coral acclimatization to changing water depth and sea surface temperature have made coral holobiont resiliency a hallmark of coral reefs ecosystems through geological time.

### Supplementary information

Supplementary Information.

Supplementary Video 1.

Supplementary Video 2.

Supplementary Video 3.

Supplementary Video 4.

Supplementary Video 5.

Supplementary Video 6.

## Data Availability

Raw, processed data and movies are available in the original source format at these following UIUC cloud data base: Main Figures and SI Figures: https://uofi.box.com/s/lmoqa7psp2ykur4m3rupxaiix29mpouz, SI Movies: https://uofi.box.com/s/pm4r3y7dg9udgnhtgwhjv3wjf4v6xf46.
