# Author Correction: Corals regulate the distribution and abundance of Symbiodiniaceae and biomolecules in response to changing water depth and sea surface temperature

**DOI:** 10.1038/s41598-023-45562-w

**Published:** 2023-11-09

**Authors:** Mayandi Sivaguru, Lauren G. Todorov, Carly A. H. Miller, Courtney E. Fouke, Cara M. O. Munro, Kyle W. Fouke, Kaitlyn E. Fouke, Melinda E. Baughman, Bruce W. Fouke

**Affiliations:** 1 Carl R. Woese Institute for Genomic Biology, University of Illinois at Urbana-Champaign, Urbana, IL USA; 2 Carl Zeiss Labs@Location Partner, Carl R. Woese Institute for Genomic Biology, University of Illinois at Urbana-Champaign, Urbana, IL USA; 3 School of Molecular and Cellular Biology, University of Illinois at Urbana-Champaign, Urbana, IL USA; 4 Department of Geology, University of Illinois at Urbana-Champaign, Urbana, IL USA; 5 Department of Biology, Denison University, Granville, OH USA; 6 Department of Ecology and Evolutionary Biology, University of California at Santa Cruz, Santa Cruz, CA USA; 7 Department of Geological Sciences, Jackson School of Geosciences, The University of Texas at Austin, Austin, TX USA; 8 The Eugene Bell Center for Regenerative Biology and Tissue Engineering, Marine Biological Laboratory, Woods Hole, MA USA; 9 Department of Evolution, Ecology and Behavior, University of Illinois at Urbana-Champaign, Urbana, IL USA; 10 Roy J. Carver Biotechnology Center, University of Illinois at Urbana-Champaign, Urbana, IL USA

Correction to: *Scientific Reports* 10.1038/s41598-021-81520-0, published online 26 January 2021

Carly A. H. Miller was omitted from the author list in the original version of this Article.

Consequently, in the Acknowledgements section,

“We also sincerely thank the Fouke Research Group at Illinois, including C.A.H. Miller for assistance in collecting field specimens and assisting MS in designing, collecting and performing immunofluorescence experiments during her graduate thesis research, as well as R. Ardisana, E. Murphy, A. Oehlert, A. Piggot, H. Vescogni, and C. Cook for providing SCUBA support, technical input and scientific discussions throughout the project.”

now reads:

“We sincerely thank the Fouke Research Group at Illinois, including R. Ardisana, E. Murphy, A. Oehlert, A. Piggot, H. Vescogni, and C. Cook for providing SCUBA support, technical input and scientific discussions throughout the project.”

And, in the Author Contributions section,

“M.S., L.G.T., C.E.F., C.M.O.M., K.W.F., K.E.F. and B.W.F. all authors provided collaborative and essential research contributions throughout the study.”

now reads:

“M.S., L.G.T., C.A.H.M., C.E.F., C.M.O.M., K.W.F., K.E.F. and B.W.F. provided collaborative and essential research contributions to the study.”

The original Article and accompanying Supplementary Information file have been corrected.

