# Supplementary material for: Corals regulate the distribution and abundance of Symbiodiniaceae and biomolecules in response to changing water depth and sea surface temperature
Source: Sci Rep. 2021 Jan 26;11:2230. doi: 10.1038/s41598-021-81520-0 (PMC7838310; doi:10.1038/s41598-021-81520-0)
Supplement: Supplementary file 1 — Supplementary Information. [file 41598_2021_81520_MOESM1_ESM.docx]

**Supplementary Figures and Captions for Sivaguru et al.,**

**Corals Regulate Tissue Distribution and Abundance of Symbiodiniaceae and Biomolecules in Response to Changing Water Depth and Sea Surface Temperature**

Mayandi Sivaguru^1,2,11*^, Lauren G. Todorov^1,3,11^, Carly A. H. Miller^1,4^, Courtney E. Fouke^1,5^, Cara M.O. Munro^1,6^, Kyle W. Fouke^1,7^, Kaitlyn E. Fouke^1,5,8^, Melinda E. Baughman^1^, and Bruce W. Fouke^1,2,4,9,10*^

^1^Carl R. Woese Institute for Genomic Biology, University of Illinois at Urbana-Champaign, Urbana, IL, USA.

^2^Carl Zeiss Labs@Location Partner, Carl R. Woese Institute for Genomic Biology University of Illinois at Urbana-Champaign, Urbana, IL, USA.

^3^School of Molecular and Cellular Biology, University of Illinois at Urbana-Champaign, Urbana, IL, USA

^4^Department of Geology, University of Illinois at Urbana-Champaign, Urbana, IL, USA.

^5^Department of Biology, Denison University, Granville, OH, USA.

^6^Department of Ecology and Evolutionary Biology, University of California at Santa Cruz, Santa Cruz, CA. USA.

^7^Department of Geological Sciences, Jackson School of Geosciences, The University of Texas at Austin, Austin, TX, USA

^8^The Eugene Bell Center for Regenerative Biology and Tissue Engineering, Marine Biological Laboratory, Woods Hole, MA, USA.

^9^Department of Evolution, Ecology and Behavior, University of Illinois at Urbana-Champaign, Urbana, IL, USA.

^10^Roy J. Carver Biotechnology Center, University of Illinois at Urbana-Champaign, Urbana, IL, USA.

^11^These authors contributed equally: Mayandi Sivaguru and Lauren G. Todorov.

**^*^Corresponding Authors:**

Mayandi Sivaguru, [sivaguru@illinois.edu](mailto:sivaguru@illinois.edu)

Bruce W. Fouke, [fouke@illinois.edu](mailto:fouke@illinois.edu)

**Supplementary Figures**


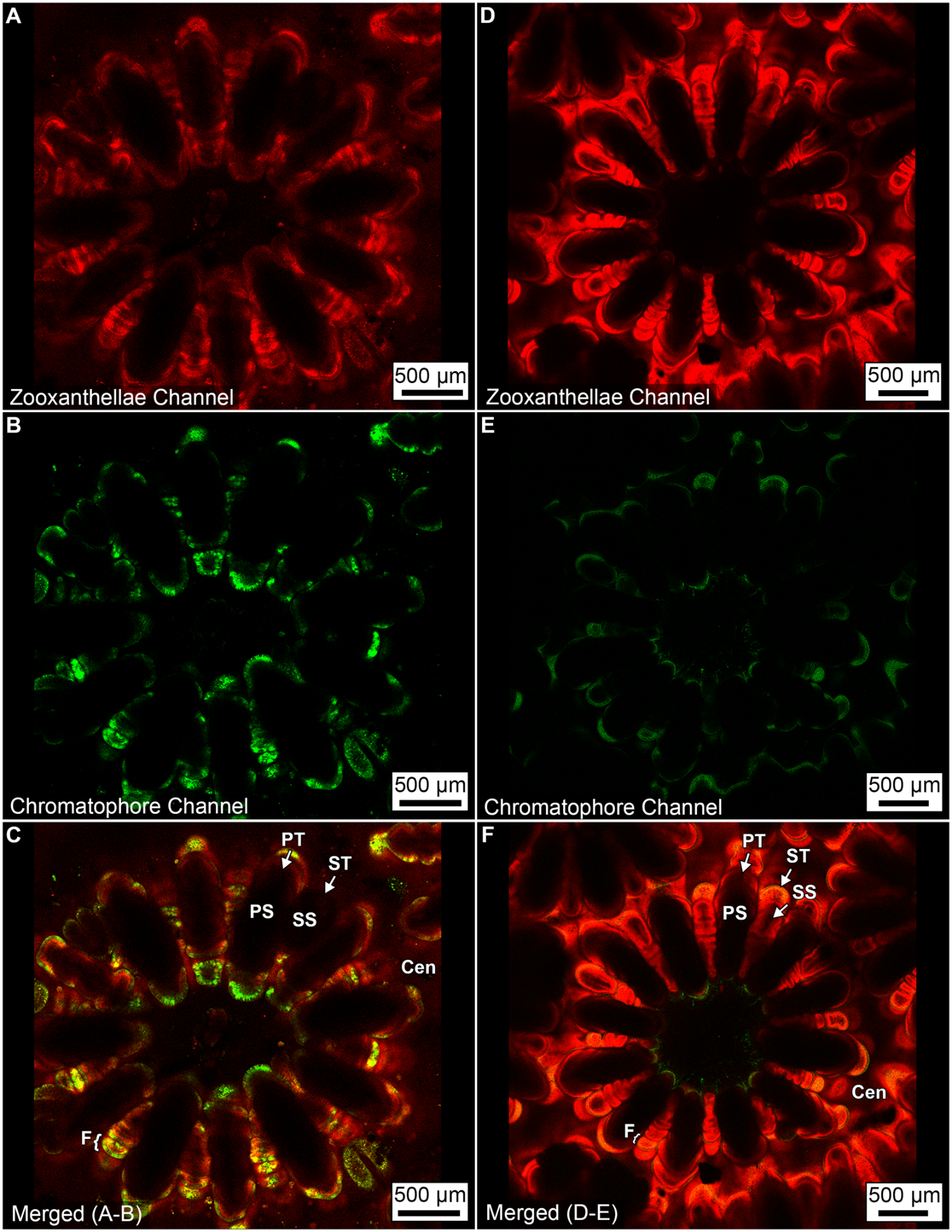


**Supplementary Figure 1. Two-Photon Laser Scanning Microscopy Showing a Single Optical Cross-Section Through the a Representative Coral Polyp Displaying the Zooxanthellae and Chromatophores of Two Reef-Building Species.** The autofluorescence from the chromatophores (pseudo-colored green) and from the chlorophyll *a* in the zooxanthellae (pseudo-colored red) were shown from a single optical section. (A) Zooxanthellae autofluorescence distribution in a single polyp of *O. annularis.* (B) Chromatophore autofluorescence distribution in a single polyp of *O. annularis.* (C) Superimposed images of A and B. (D) Zooxanthellae autofluorescence distribution in a single polyp of *O. faveolata.* (E) Chromatophore autofluorescence distribution in a single polyp of *O. faveolata.* (F) Superimposed images of D and E*.* Labeled components in images include: Cen, ceonosarc; PS, primary septum; SS, secondary septum; PT, primary tentacle tip; ST, secondary tentacle tip; F, a single fold in a set of folded polyp walls within a septum.


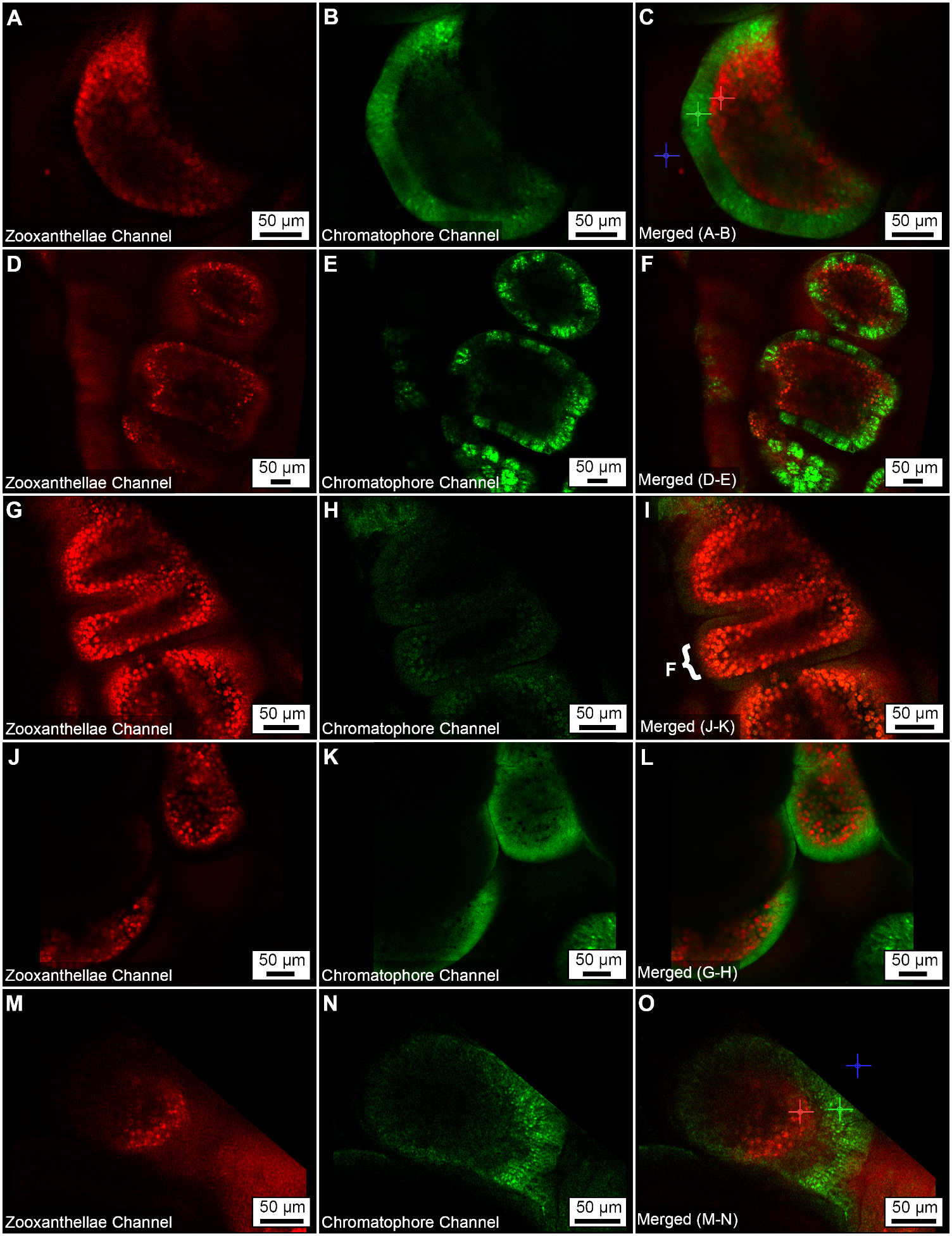


**Supplementary Figure 2. High Magnification Two-Photon Images of a Representative Single Optical Cross-Section Through Coral Tissue at Various Locations Showing Zooxanthellae (red) and Chromatophores (green).** The autofluorescence from the chromatophores (pseudo-colored green) and from the chlorophyll *a* in the zooxanthellae (pseudo-colored red) were shown from a single optical section. (A-C) One fold of the polyp wall tissue overlying a primary septum in *O. annularis* excited by a single-photon 405 nm wavelength of light and emission collected at green (500-550 nm) and red (575-615 nm) wavelength bands. (D-F) Folded polyp wall tissues and tentacles overlying a primary septum in *O. annularis* excited by two-photon 780 nm wavelength of light and emission collected at green (500-550 nm) and red (575-615 nm) wavelength bands. (G-I) Folded polyp wall tissues and tentacles overlying a primary septum in *O. faveolata* excited by two-photon 780 nm wavelength of light and emission collected at green (500-550 nm) and red (575-615 nm) wavelength bands*.* (J-L) The tip of a tentacle overlying a secondary septum in *O. faveolata* excited by a single-photon 405 nm wavelength of light and emission collected at green (500-550 nm) and red (575-615 nm) wavelength bands. (M-O) The tip of a tentacle overlying a primary septum in *O. faveolata* excited by two-photon 780 nm wavelength of light and emission collected at green (500-550 nm) and red (575-615 nm) wavelength bands. Note that the tip of the tentacles on the secondary septa in *O. annularis* do not have noticeable zooxanthellae or chromatophores, therefore it was not included in this figure. Spectral data for identification is derived from the points labeled with colored crosses: red for zooxanthellae, green for chromatophores, and blue for the background. Relative intensities of all images were adjusted to the entire plate for better visualization and comparison. Labeled component in images include: F, a single fold in a set of folded polyp walls within a septum.

**
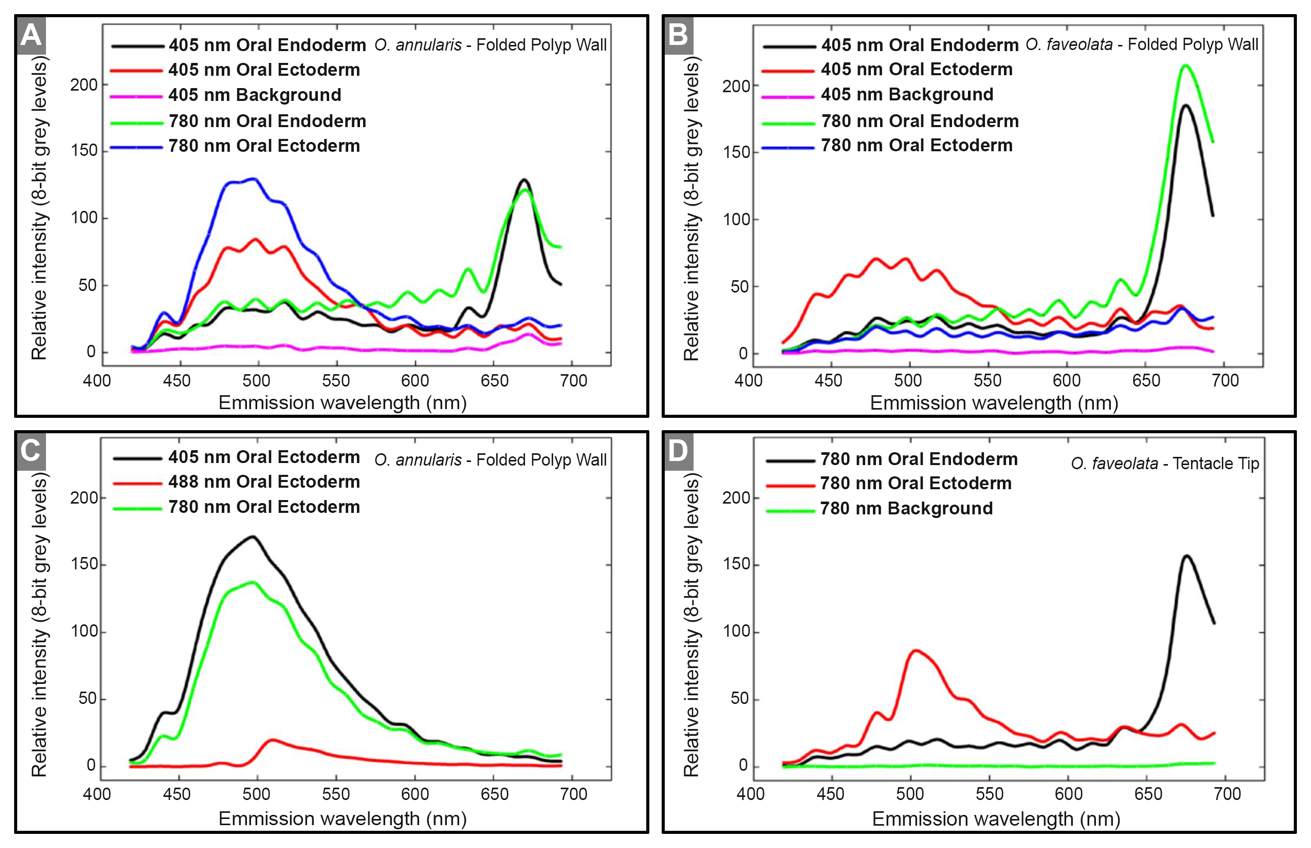
**

**Supplementary Figure 3. Single and Two-Photon Confocal Spectral Analysis of *O. annularis* and *O. faveolata* Specific Tissue Locations as Shown in Supplementary Figure 2.** Coral species, specific tissue location and excitation wavelength used collected throughout the visible spectrum (collected between 400-700 nm at ~10 nm intervals using spectral detector) are displayed in A-D. Wavelengths are color-coded*. O. annularis* and *O. faveolata* tissue was excited by 405 nm (UV-A/blue light; single photon) and 780 nm (red laser light; two-photon) wavelengths. In addition to 405 nm and 780 nm wavelengths of light, the tested single-photon 488 nm excitation failed to produce the peak observed ~490 nm in the folded polyp wall by the other wavelengths, indicating the necessity of using the combination of 405 nm and 780 nm wavelengths. The autofluorescence emission peak from chlorophyll *a* in zooxanthellae cells is at ~670 nm. The autofluorescence emission peak from the chromatophore cells, GFP-like compound, is at ~490 nm. When a component in the tissue at the given location is non-responsive to a given excitation wavelength, then there will be no peaks observed. Note the background spectra are obtained outside the tissue region observed as black in Supplementary Figure. 2C and O.


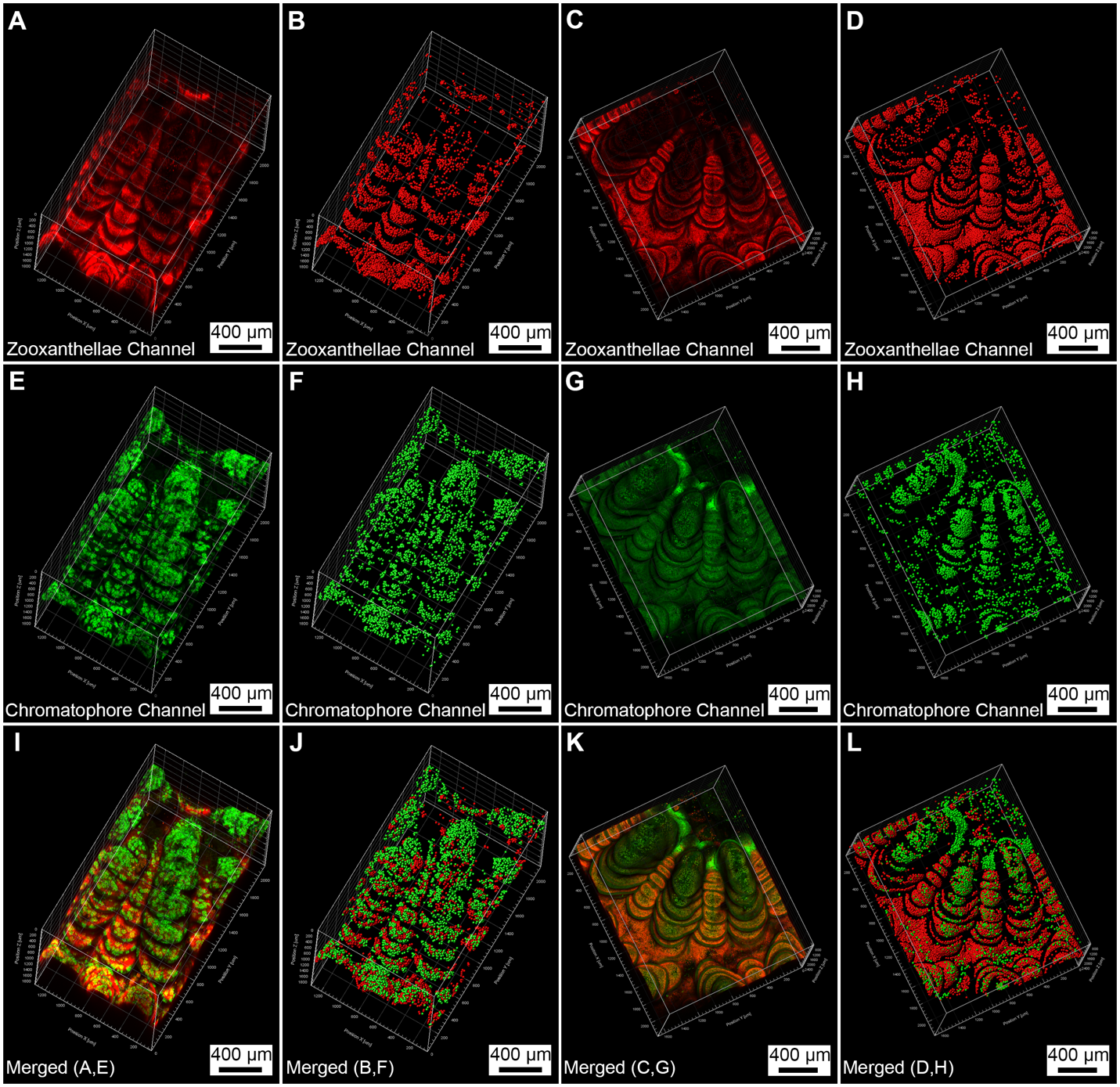


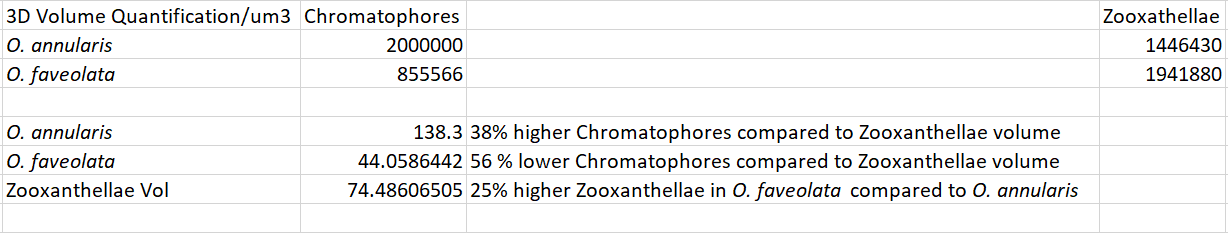


**Supplementary Figure 4. Three-Dimensional Volumetric Quantification of Zooxanthellae and Chromatophores of Representative Segment of a Coral Polyp from Two Reef-Building Species.** The autofluorescence from the chromatophores (pseudo-colored green) and from the chlorophyll *a* in the zooxanthellae (pseudo-colored red) were 3D reconstructed from optical sections displayed in raw (A, E, I, C, G, K) and rendered in 3D after spot quantification (B, F, J, D, H, L). (A) The zooxanthellae autofluorescence distribution of *O. annularis.* (B) 3D-rendered image of A after spot quantification by the Imaris 3D-spot Isosurface rendering algorithm*.* (C) The zooxanthellae autofluorescence distribution of *O. faveolata.* (D) 3D-rendered image of C after spot quantification by the Imaris 3D-spot Isosurface rendering algorithm*.* (E) The chromatophore autofluorescence distribution of *O. annularis.* (F) 3D-rendered image of E after spot quantification by the Imaris 3D-spot Isosurface rendering algorithm*.* (G) The chromatophore autofluorescence distribution of *O. faveolata.* (H) 3D-rendered image of G after spot quantification by the Imaris 3D-spot Isosurface rendering algorithm*.* (I) Merged images of A and E (See also Supplementary Movie 3). (J) 3D-rendered image of I after spot quantification by the Imaris 3D-spot Isosurface rendering algorithm (See also Supplementary Movie 4)*.* (K) Merged images of B and F (See also Supplementary Movie 5). (L) 3D-rendered image of K after spot quantification by the Imaris 3D-spot Isosurface rendering algorithm (See also Supplementary Movie 6)*.* Quantification of 3D spot data (converted to 2D) is analyzed from individual tissue overlying primary and secondary septa are performed following the strategies described in Supplementary Figure 11 and the data presented in Figure 5A-B. The table below the image shows the 3D volume quantification based on spot count data in the Imaris program.

**
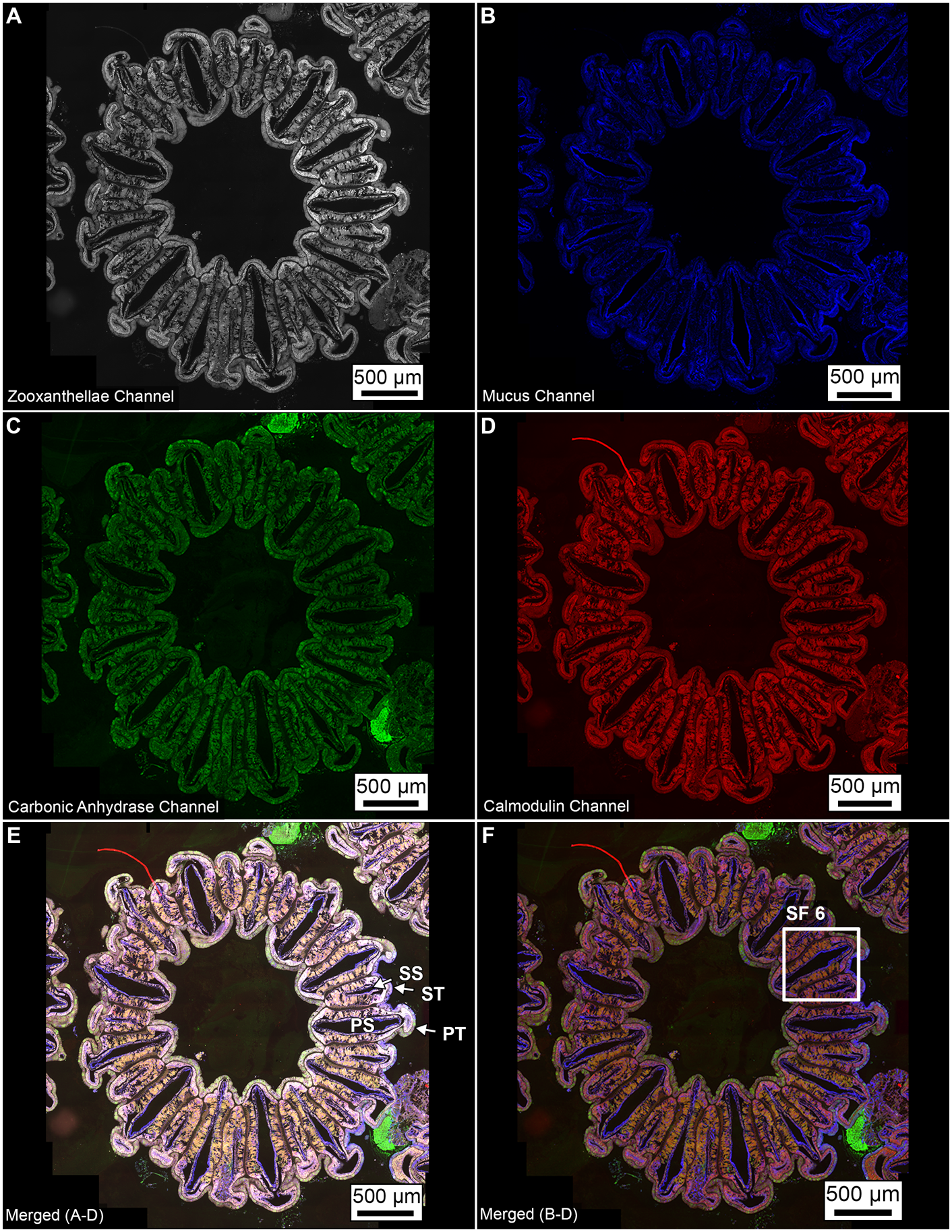
**

**Supplementary Figure 5. Qualitative and Quantitative Analyses of the Cellular and Molecular Components (Presented with Individual Channels) of *O. faveolata* Tissue in March 2008 Image Displayed in Figure 6A.** (A) The zooxanthellae autofluorescence displayed pseudo-colored white. (B) The WGA-labeled mucus displayed pseudo-colored blue. (C) The carbonic anhydrase (CA) tagged by a custom-made primary antibody pseudo-colored green. (D) Calmodulin tagged by antibody 6D4 is pseudo-colored red. (E) Merged images of A-D. (F) Merged images of B-D. The white zooxanthellae channel was removed for optimal fluorescence visualization. The overlap of the three colored light filters (blue, green, and red) shift the observed color in varying degrees. The equal overlap of the green and red channels exhibit yellow-orange, green and blue display cyan, red and blue express magenta, while the combination of all three is observed as white. If a red and blue channel merged image contains a stronger emission of red compared to blue at a given location, pink is detected. On the other hand, if the blue light channel emission is higher in intensity relative to the red, then a deep purple color is seen. Note that that the bright green stains and red line are preparation artifacts. Hence, the relative intensity of each color represents the extent of the presence of a given molecular component. White boxes indicated the selected locations for Supplementary Figure 6. Labeled components in images include: PS, primary septum; SS, secondary septum; PT, primary tentacle tip; ST, secondary tentacle tip.

**
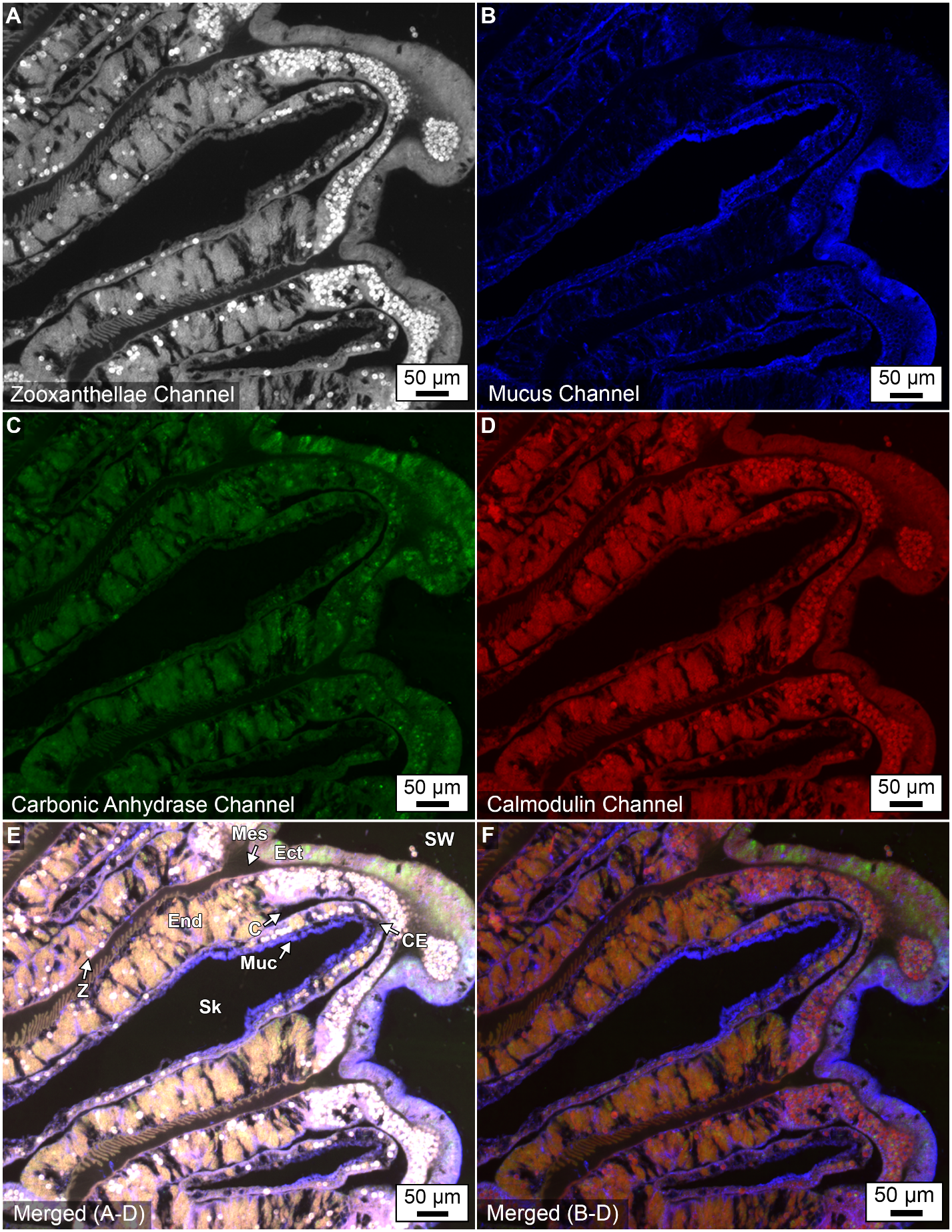
**

**Supplementary Figure 6. High Magnification Images Showing Analysis of the Cellular and Molecular Components (Presented with Individual Channels) of a Representative Region of Interest from an *O. faveolata* Coral Polyp Obtained in March 2008.** The image is a selected region highlighted in Supplementary Figure 5F. (A) The zooxanthellae autofluorescence displayed pseudo-colored white. (B) The WGA-labeled mucus displayed pseudo-colored blue. (C) The carbonic anhydrase (CA) tagged by a custom-made primary antibody pseudo-colored green. (D) Calmodulin tagged by antibody 6D4 is pseudo-colored red. (E) Merged images of A-D. Merged images of B-D. The white zooxanthellae channel was removed for optimal fluorescence visualization. The overlap of the three colored light filters (blue, green, and red) shift the observed color in varying degrees. The equal overlap of the green and red channels exhibit yellow-orange, green and blue display cyan, red and blue express magenta, while the combination of all three is observed as white. If a red and blue channel merged image contains a stronger emission of red compared to blue at a given location, pink is detected. On the other hand, if the blue light channel emission is higher in intensity relative to the red, then a deep purple color is seen. Note that that the bright green stains and red line are preparation artifacts. Hence, the relative intensity of each color represents the extent of the presence of a given molecular component. Labeled components in images include: SW, sea water; ; Sk, skeleton; Ect, oral ectoderm; Mes, mesoglea; End, oral endoderm; Z, zooxanthellae; Coe, coelenteron; CE, calicoblastic epithelium.


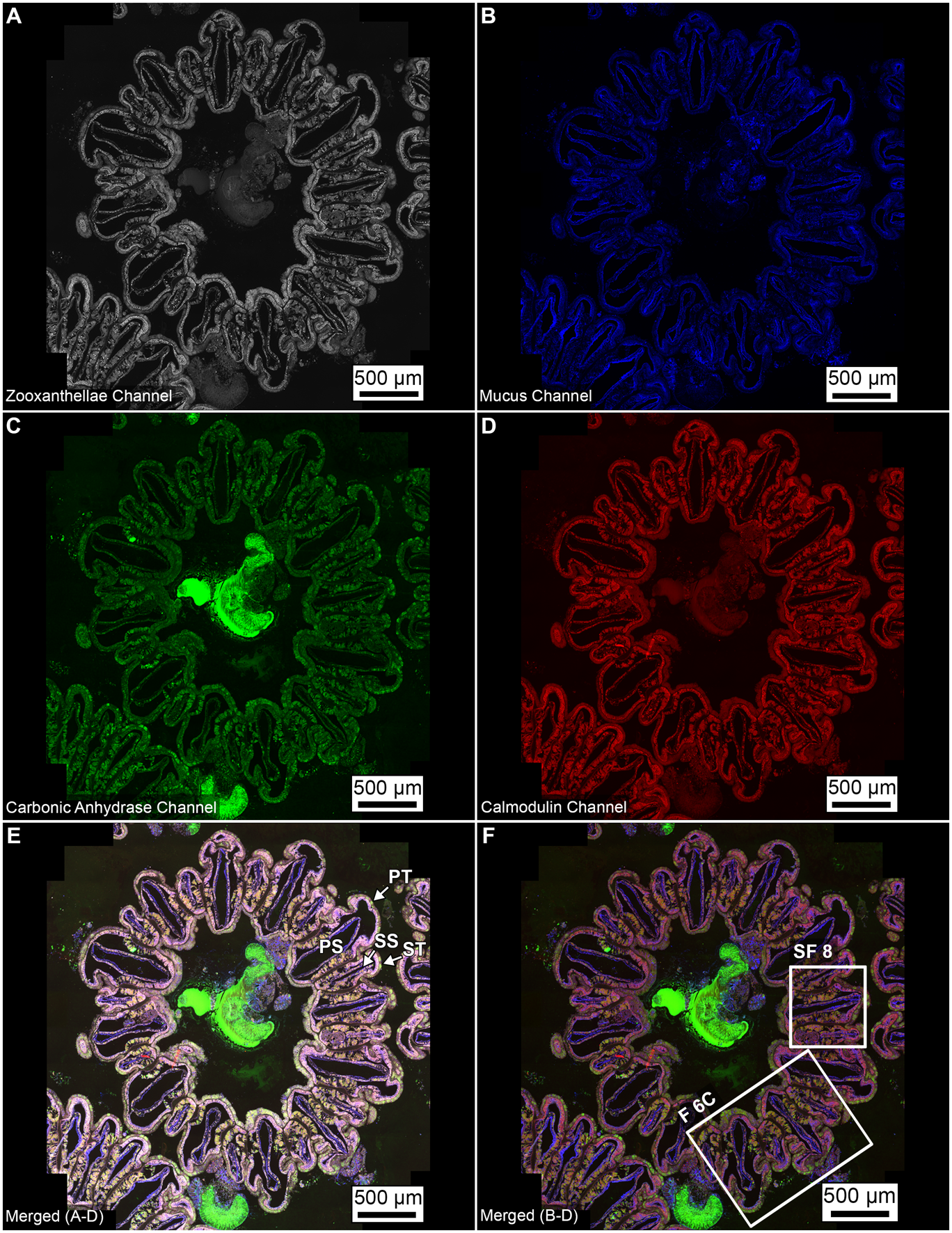


**Supplementary Figure 7. Qualitative and Quantitative Analyses of the Cellular and Molecular Components (Presented with Individual Channels) of *O. faveolata* Tissue in May 2008 Image Displayed in Figure 7B.** (A) The zooxanthellae autofluorescence displayed pseudo-colored white. (B) The WGA-labeled mucus displayed pseudo-colored blue. (C) The carbonic anhydrase (CA) tagged by a custom-made primary antibody pseudo-colored green. (D) Calmodulin tagged by antibody 6D4 is pseudo-colored red. (E) Merged images of A-D. (F) Merged images of B-D. The white zooxanthellae channel was removed for optimal fluorescence visualization. The overlap of the three colored light filters (blue, green, and red) shift the observed color in varying degrees. The equal overlap of the green and red channels exhibit yellow-orange, green and blue display cyan, red and blue express magenta, while the combination of all three is observed as white. If a red and blue channel merged image contains a stronger emission of red compared to blue at a given location, pink is detected. On the other hand, if the blue light channel emission is higher in intensity relative to the red, then a deep purple color is seen. Bright green in the middle is an unspecific binding of secondary antibody. Hence, the relative intensity of each color represents the extent of the presence of a given molecular component. White boxes indicated the selected locations for Figure 6C and Supplementary Figure 8. Labeled components in images include: PS, primary septum; SS, secondary septum; PT, primary tentacle tip; ST, secondary tentacle tip.

**
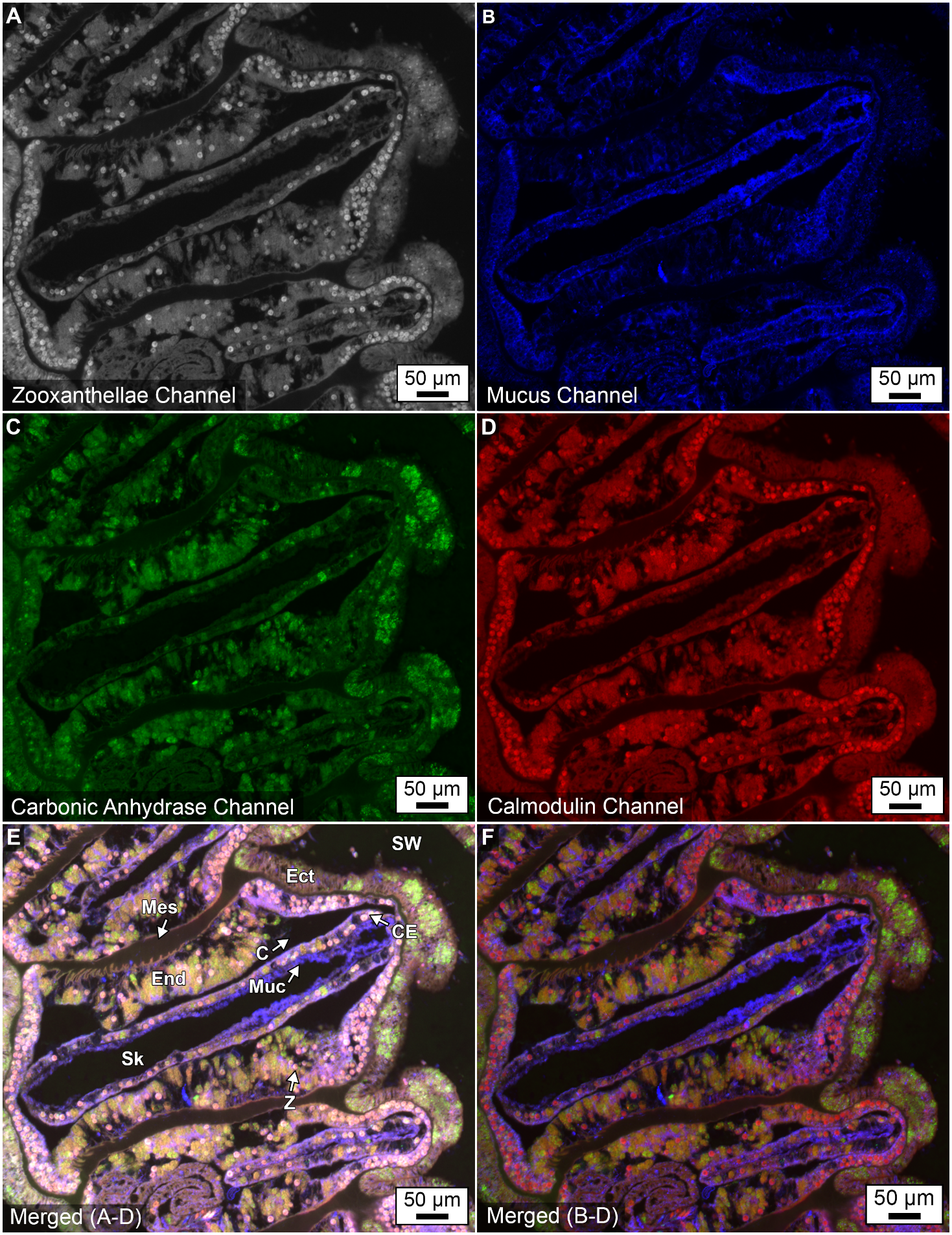
**

**Supplementary Figure 8. High Magnification Images Showing Analysis of the Cellular and Molecular Components (Presented with Individual Channels) of a Representative Region of Interest from an *O. faveolata* Coral Polyp Obtained in May 2008.** The image is a selected region highlighted in Figure 7A-B and Supplementary Figure 7F. (A) The zooxanthellae autofluorescence displayed pseudo-colored white. (B) The WGA-labeled mucus displayed pseudo-colored blue. (C) The carbonic anhydrase (CA) tagged by a custom-made primary antibody pseudo-colored green. (D) Calmodulin tagged by antibody 6D4 is pseudo-colored red. (E) Merged images of A-D. (F) Merged images of B-D. The white zooxanthellae channel was removed for optimal fluorescence visualization. The overlap of the three colored light filters (blue, green, and red) shift the observed color in varying degrees. The equal overlap of the green and red channels exhibit yellow-orange, green and blue display cyan, red and blue express magenta, while the combination of all three is observed as white. If a red and blue channel merged image contains a stronger emission of red compared to blue at a given location, pink is detected. On the other hand, if the blue light channel emission is higher in intensity relative to the red, then a deep purple color is seen. Hence, the relative intensity of each color represents the extent of the presence of a given molecular component. Labeled components in images include: SW, sea water; Sk, skeleton; Ect, oral ectoderm; Mes, mesoglea; End, oral endoderm; Z, zooxanthellae; C, coelenteron; CE, calicoblastic epithelium.


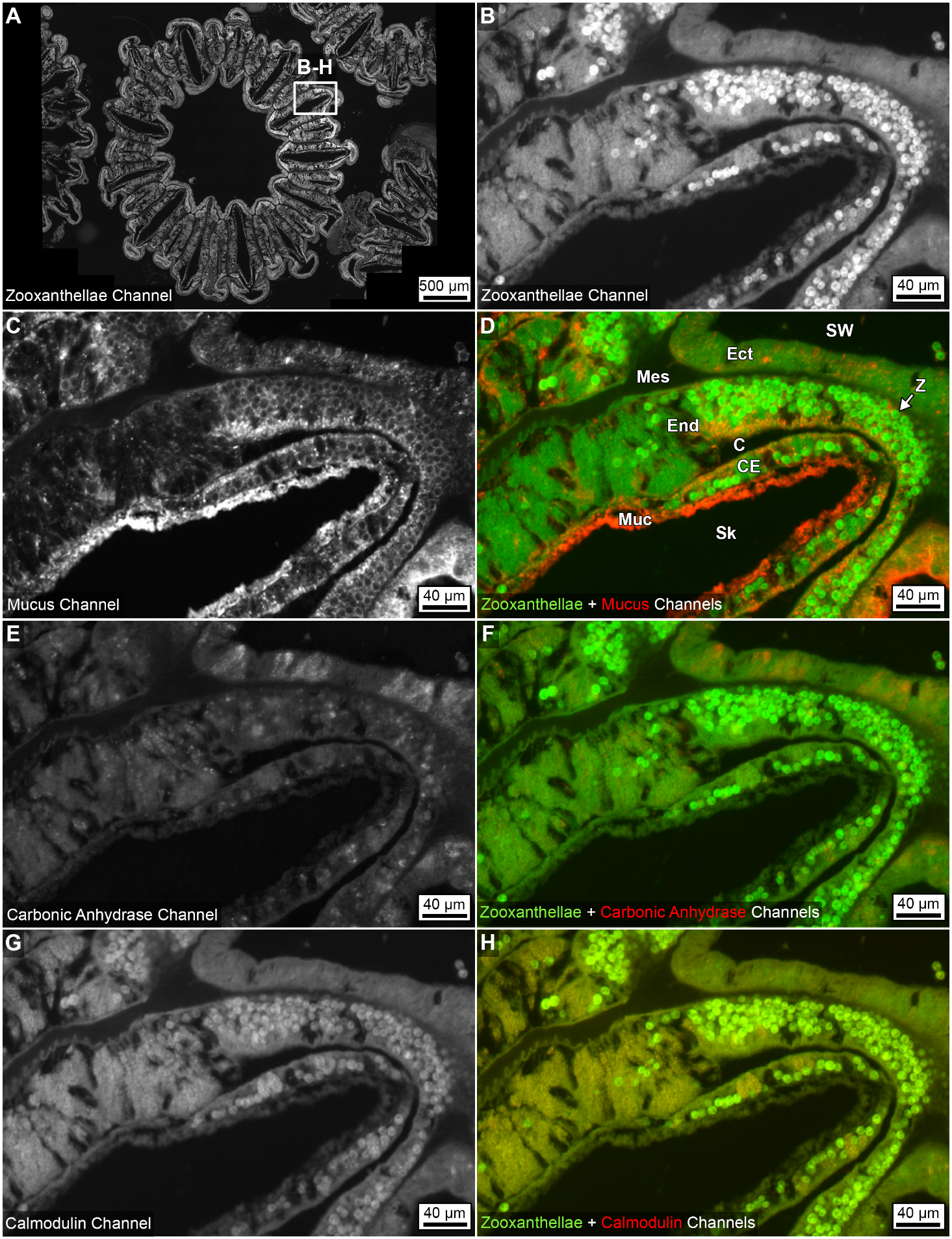


**Supplementary Figure 9. Qualitative and Quantitative Analyses of the Cellular and Molecular Components (Presented with Individual Channels) of *O. faveolata* Tissue in March 2008 Image Displayed in Supplementary Figure 6.** (A) Contextual image displaying the whole polyp with the zooxanthellae channel presented in gray-scale. (B) Enlargement of the tissue overlying the primary septa with the zooxanthellae autofluorescence presented in gray-scale. (C) The WGA-labeled mucus displayed presented in gray-scale. (D) The zooxanthellae autofluorescence displayed in pseudo-colored green overlaid on the WGA-labeled mucus displayed in pseudo-colored red. (E) The carbonic anhydrase (CA) tagged by a custom-made primary antibody presented in gray-scale. (F) The zooxanthellae autofluorescence channel displayed in green overlaid on the CA tagged by a custom-made primary antibody displayed in red. (G) Calmodulin tagged by antibody 6D4 fluorescence presented in gray-scale. (H) The zooxanthellae autofluorescence channel displayed in green overlaid on the calmodulin tagged by antibody 6D4 fluorescence in red. The overlap of the green and red channels exhibit yellow when the individual channels are equal in intensity. If the red channel is higher in intensity relative to the green channel than an orange color is observed. Labeled components in images include: SW, sea water; Sk; skeleton; Ect, oral ectoderm; Mes, mesoglea; End, oral endoderm; Z, zooxanthellae; C, coelenteron; CE, calicoblastic epithelium.


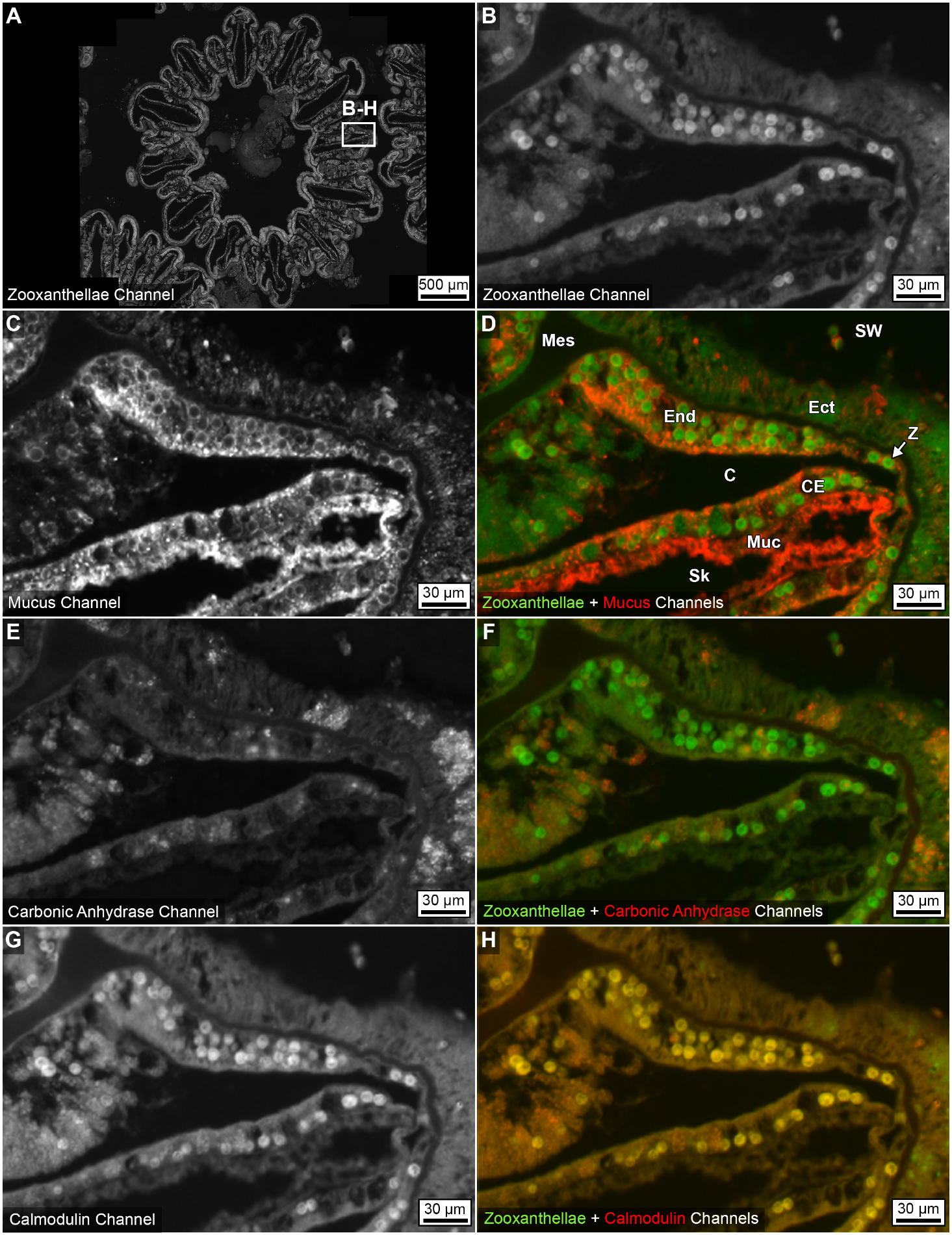


**Supplementary Figure 10. Qualitative and Quantitative Analyses of the Cellular and Molecular Components (Presented with Individual Channels) of *O. faveolata* Tissue in May 2008 Image Displayed in Supplementary Figure 8.** (A) Contextual image displaying the whole polyp with the zooxanthellae channel presented in gray-scale. (B) Enlargement of the tissue overlying the primary septa with the zooxanthellae autofluorescence presented in gray-scale. (C) The WGA-labeled mucus displayed presented in gray-scale. (D) The zooxanthellae autofluorescence displayed in pseudo-colored green overlaid on the WGA-labeled mucus displayed in pseudo-colored red. (E) The carbonic anhydrase (CA) tagged by a custom-made primary antibody presented in gray-scale. (F) The zooxanthellae autofluorescence channel displayed in green overlaid on the CA tagged by a custom-made primary antibody displayed in red. (G) Calmodulin tagged by antibody 6D4 fluorescence presented in gray-scale. (H) The zooxanthellae autofluorescence channel displayed in green overlaid on the calmodulin tagged by antibody 6D4 fluorescence in red. The overlap of the green and red channels exhibit yellow when the individual channels are equal in intensity. If the red channel is higher in intensity relative to the green channel than an orange color is observed. Labeled components in images include: SW, sea water; Sk, skeleton; Ect, oral ectoderm; Mes, mesoglea; End, oral endoderm; Z, zooxanthellae; Coe, coelenteron; CE, calicoblastic epithelium.


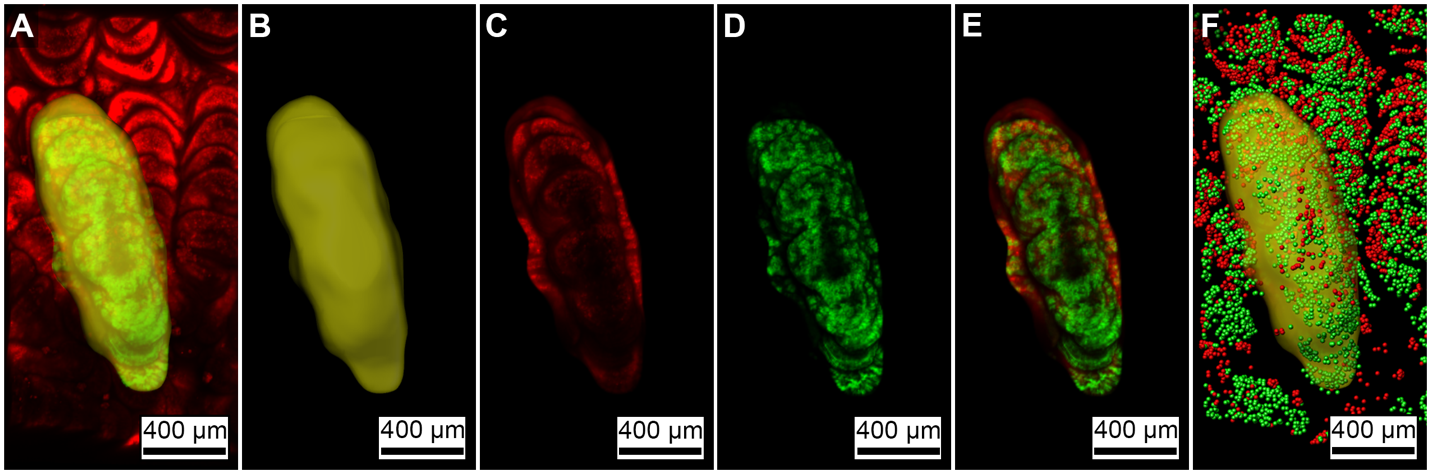


**Supplementary Figure 11. Three-Dimensional Volumetric Quantification Method of Zooxanthellae and Chromatophores Distribution within a Representative Segment of the Tissue Overlying the Primary Septa of a Single Coral Polyp.** Note that the tissue overlying the secondary septa from the same coral polyp of images presented Supplementary Figure 4 was also quantified using the same strategy as displayed above for the tissue overlying the primary septa. (A) A contour of a given tissue overlying the primary or secondary septa from a series of optical sections have been obtained by manually tracing the contour. (B) Traced contour surface contains the 3D region of interest for zooxanthellae and chromatophore quantification. (C) The zooxanthellae distribution from the raw data was extracted from the 3D trace contour surface area. (D) The chromatophore distribution from the raw data was extracted from the 3D trace contour surface area. (E) Merge of C and D. (F) The raw data in E was 3D-rendered by the Imaris 3D-spot Isosurface rendering algorithm*.* Since the individual 3D region of tissue overlying the single primary or secondary septa could be obtained this way, the quantification of spot in 3D is only obtained within the contour surface contain the region of interest.

**
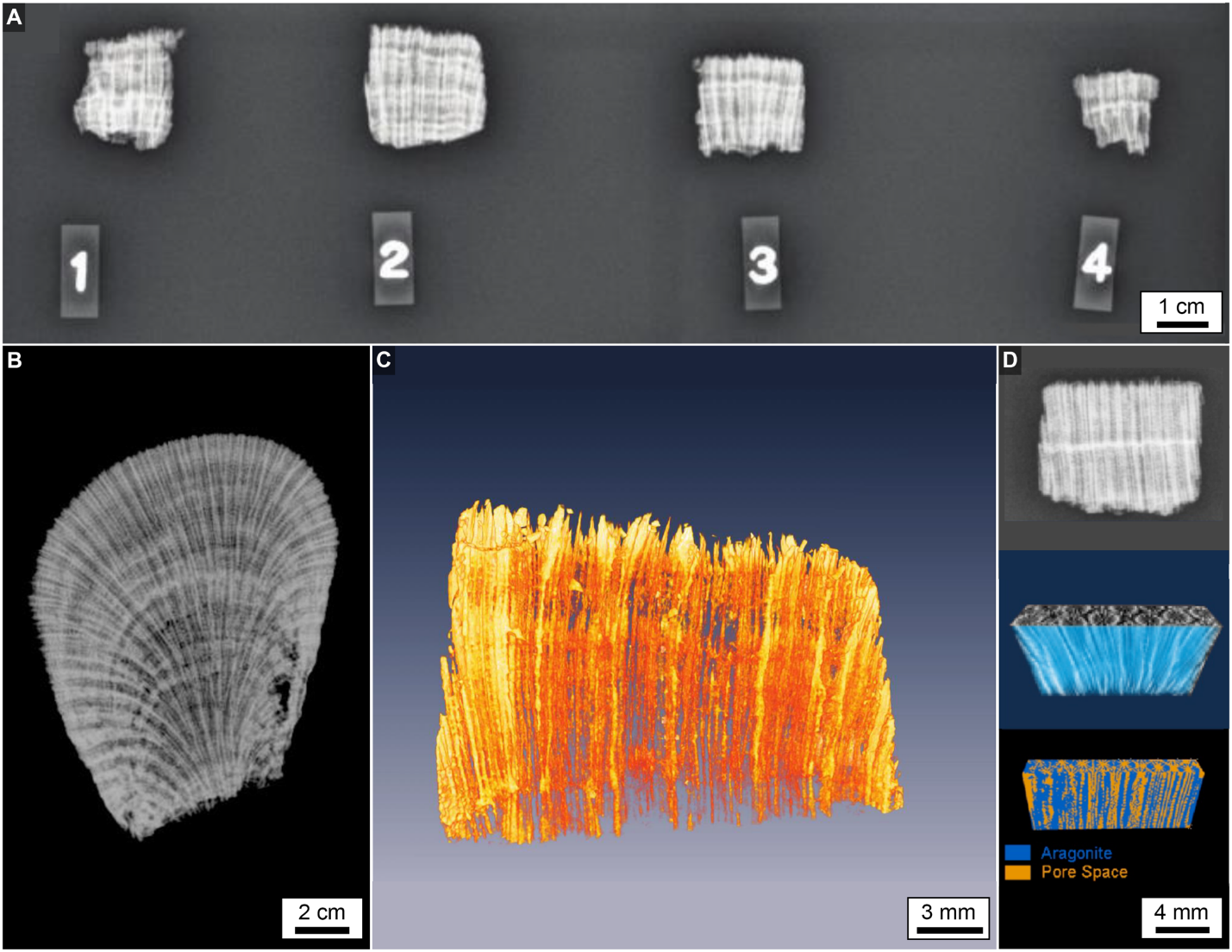
**

**Supplementary Figure 12. Coral skeletal density banding in *O. faveolata* in skeletal biospies.** (A) X-radiographs of four 1-2 cm-thick skeletal biopsies showing the distribution of HDB and LDB layers. (B) X-radiograph of an entire small head of *O. annularis* showing HDB and LDB layers. Note that in both A and B, the darker regions are LDB and brighter regions are HDB. (C) High resolution microCT enlargement of Sample 2 in A showing horizontal HDB and LDB layers. (D) Example of how HDB and LDB layer segmentation was performed to select individual horizons from 5 mm-thick 3D microCT scans virtual slices. These quantitative analyses of the volume percent aragonite skeleton and volume percent porosity are reported in Fig. 8B.

**Supplementary Movie Captions**


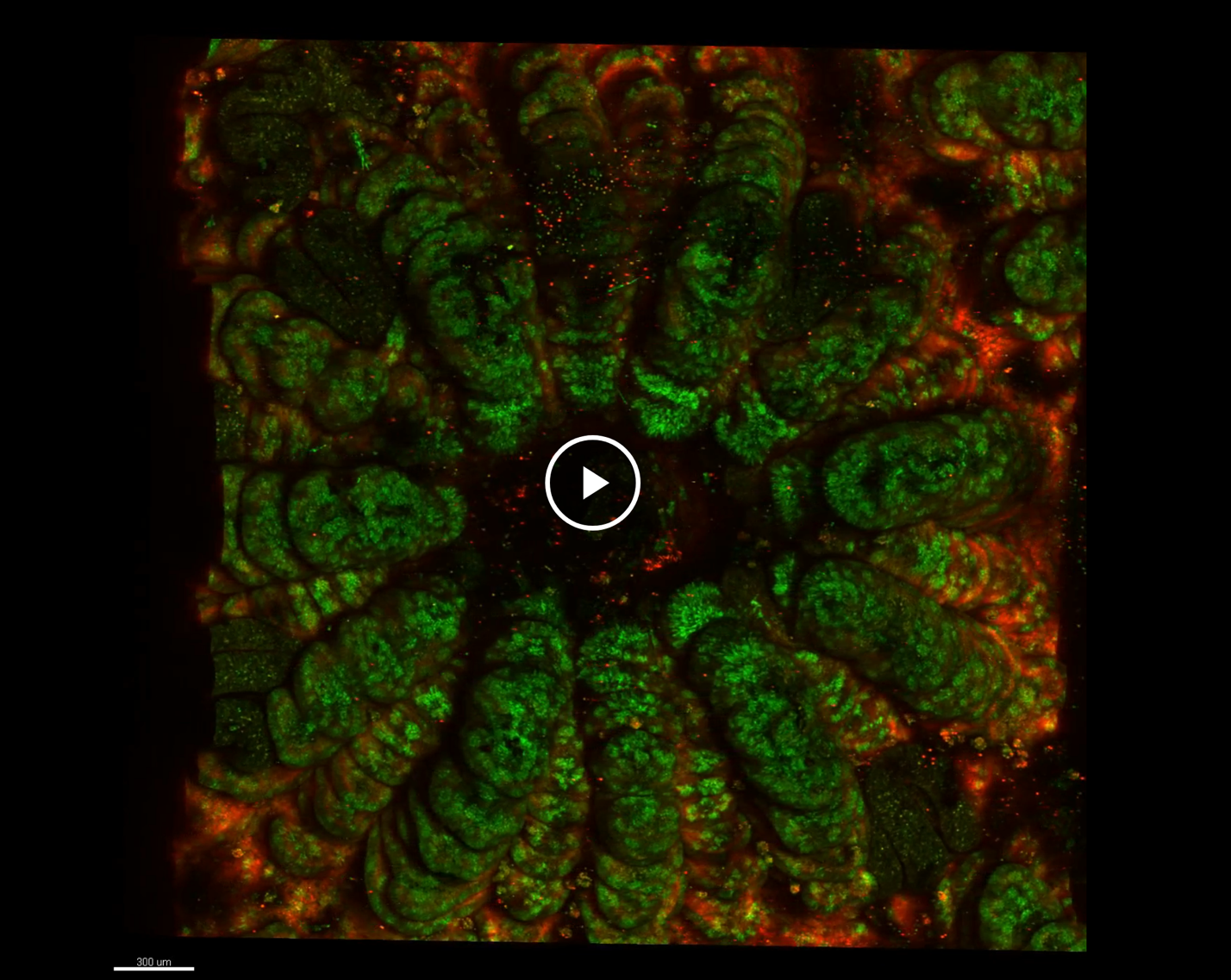


**Supplementary Movie 1. Two-Photon Laser Scanning Microscopy Showing Three-Dimensional Reconstruction of a Representative Coral Polyp Displaying the Zooxanthellae and Chromatophores of *O. annularis* as Illustrated in Figure 4C.** 3D raw data two-photon microscopy image of the coral polyp *O. annularis*. The excitation is 780 nm and the emission captured simultaneously at two band widths for zooxanthellae (pseudo-colored red, 600-700 nm) and chromatophores (pseudo-colored green, 500-550 nm). This movie displays an aerial and profile topographic view of the coral polyp. Additionally, the individual channel for zooxanthellae and chromatophore autofluorescence are displayed independently and merged. Lastly, the movie displays optical sectioning from the top to the bottom, followed by the bottom to the top.


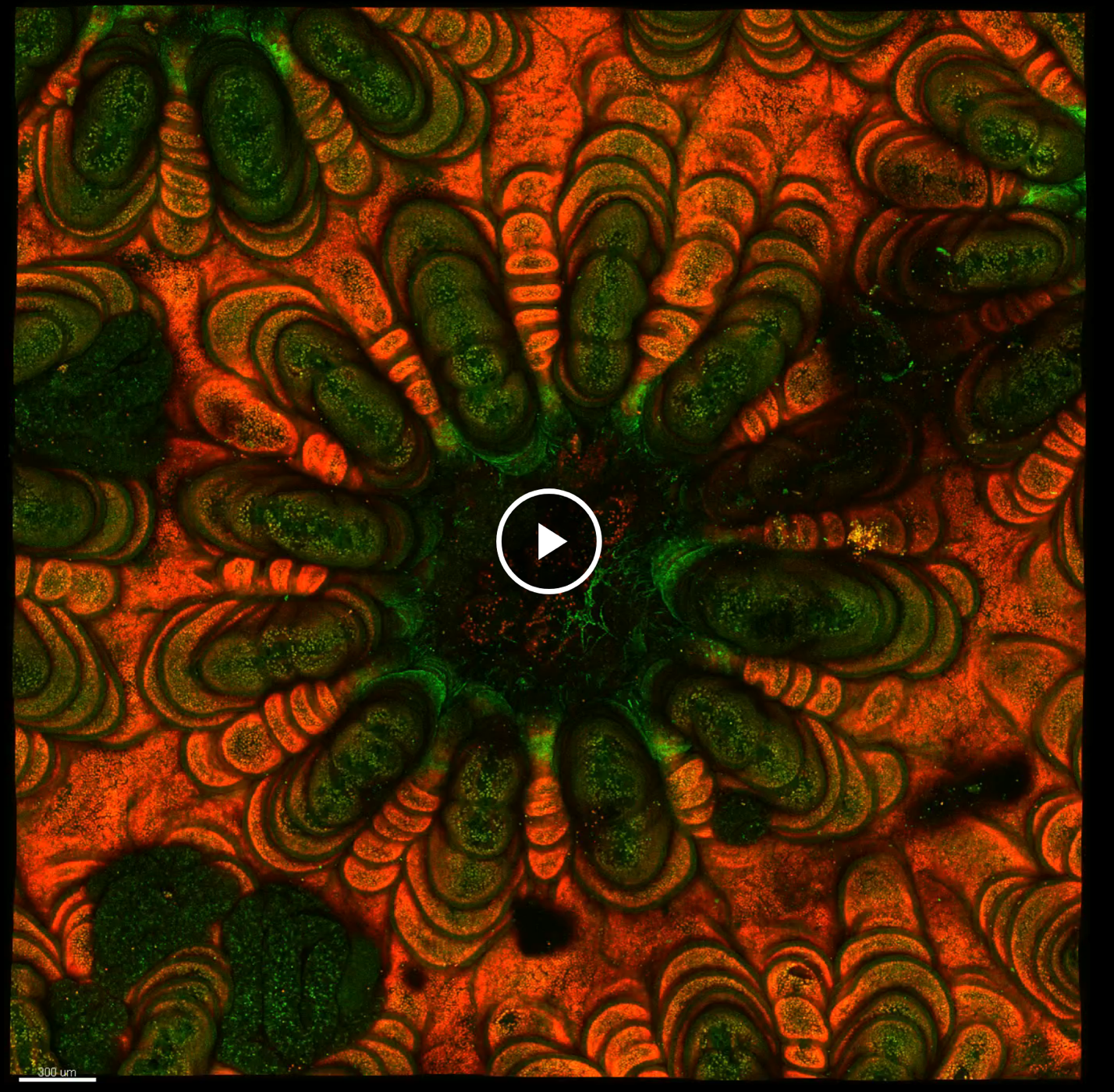


**Supplementary Movie 2. Two-Photon Laser Scanning Microscopy Showing Three-Dimensional Reconstruction of a Representative Coral Polyp Displaying the Zooxanthellae and Chromatophores of *O. faveolata* as Illustrated in Figure 4F.** 3D raw data two-photon microscopy image of the coral polyp *O. faveolata*. The excitation is 780 nm and the emission captured simultaneously at two band widths for zooxanthellae (pseudo-colored red, 600-700 nm) and chromatophores (pseudo-colored green, 500-550 nm). This movie displays an aerial and profile topographic view of the coral polyp. Additionally, the individual channel for zooxanthellae and chromatophore autofluorescence are displayed independently and merged. Lastly, the movie displays optical sectioning from the top to the bottom, followed by the bottom to the top.


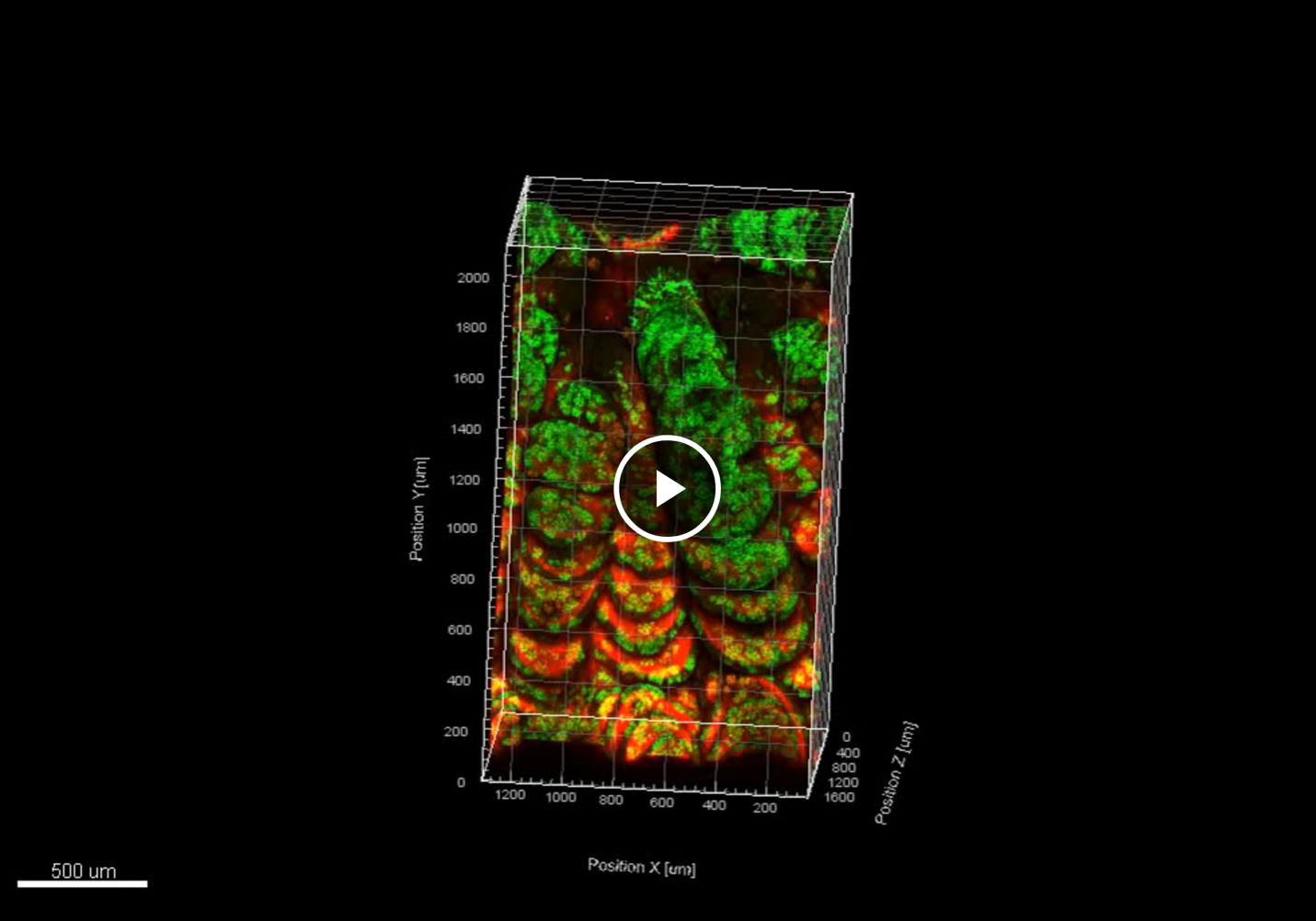


**Supplementary Movie 3. Three-Dimensional Volumetric Quantification of Zooxanthellae and Chromatophores of Representative Segment of a Coral Polyp from *O. annularis*.** 3D raw data two-photon microscopy image of the coral polyp displayed in the Supplementary Figure 4I. The excitation is 780 nm and the emission captured simultaneously at two band widths for zooxanthellae (pseudo-colored red, 600-700 nm) and chromatophores (pseudo-colored green, 500-550 nm). This movie displays an aerial and profile topographic view from a region of the folded polyp wall of the coral. The regions included in this movie are two tissues overlying the primary and secondary septa. The image is rendered inside a 3D grid with a scale.


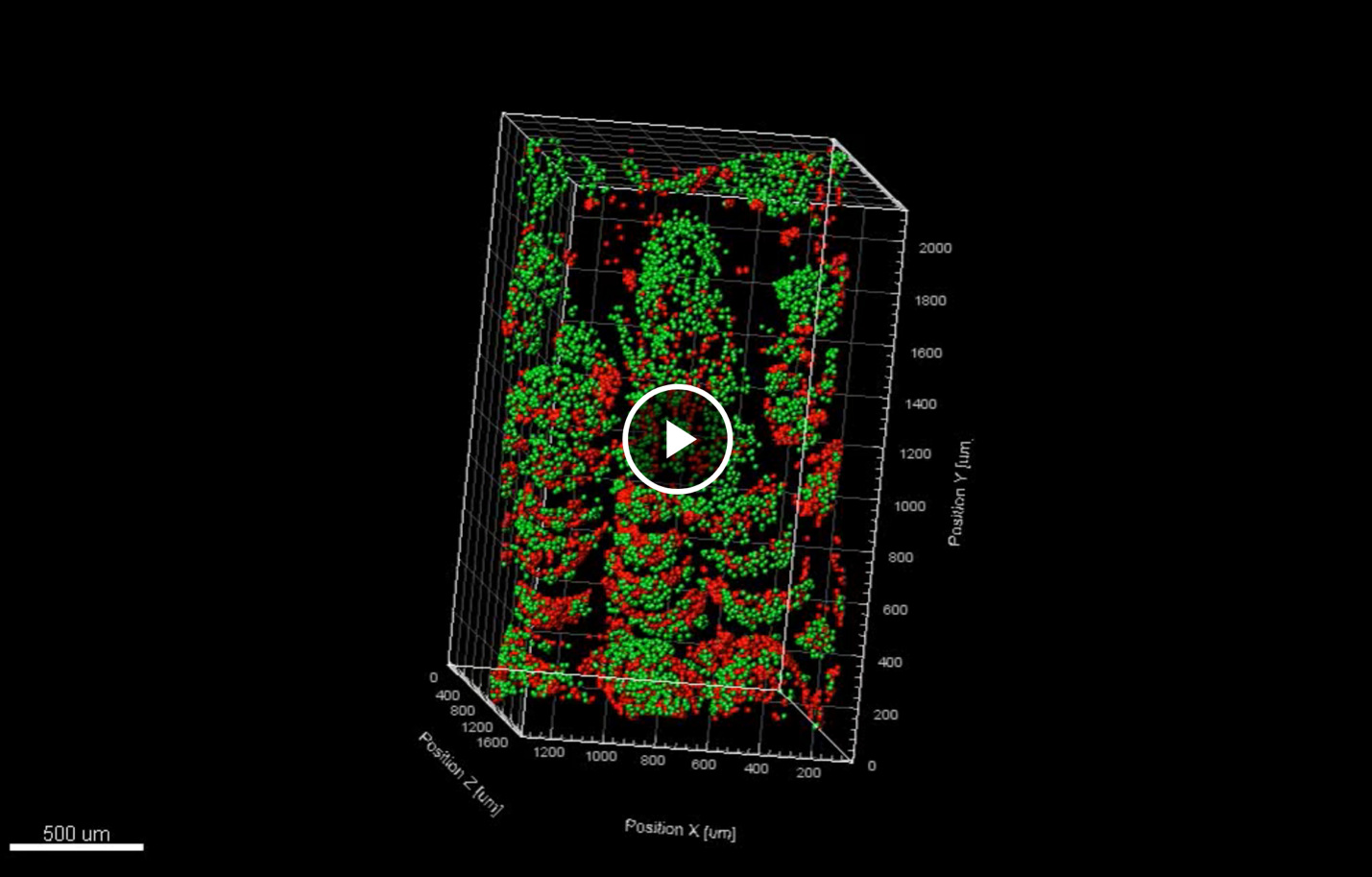


**Supplementary Movie 4. Three-Dimensional Volumetric Quantification of Zooxanthellae and Chromatophores of Representative Segment of a Coral Polyp from *O. annularis*.** 3D-rendered image of Supplementary Figure 4I and Supplementary Movie 3 after spot quantification by the Imaris 3D-spot Isosurface rendering algorithm. The excitation is 780 nm and the emission captured simultaneously at two band widths for zooxanthellae (pseudo-colored red, 600-700 nm) and chromatophores (pseudo-colored green, 500-550 nm). This movie displays an aerial and profile topographic view from a region of the folded polyp wall of the coral. The regions included in this movie are two tissues overlying the primary and secondary septa. The image is rendered inside a 3D grid with a scale.


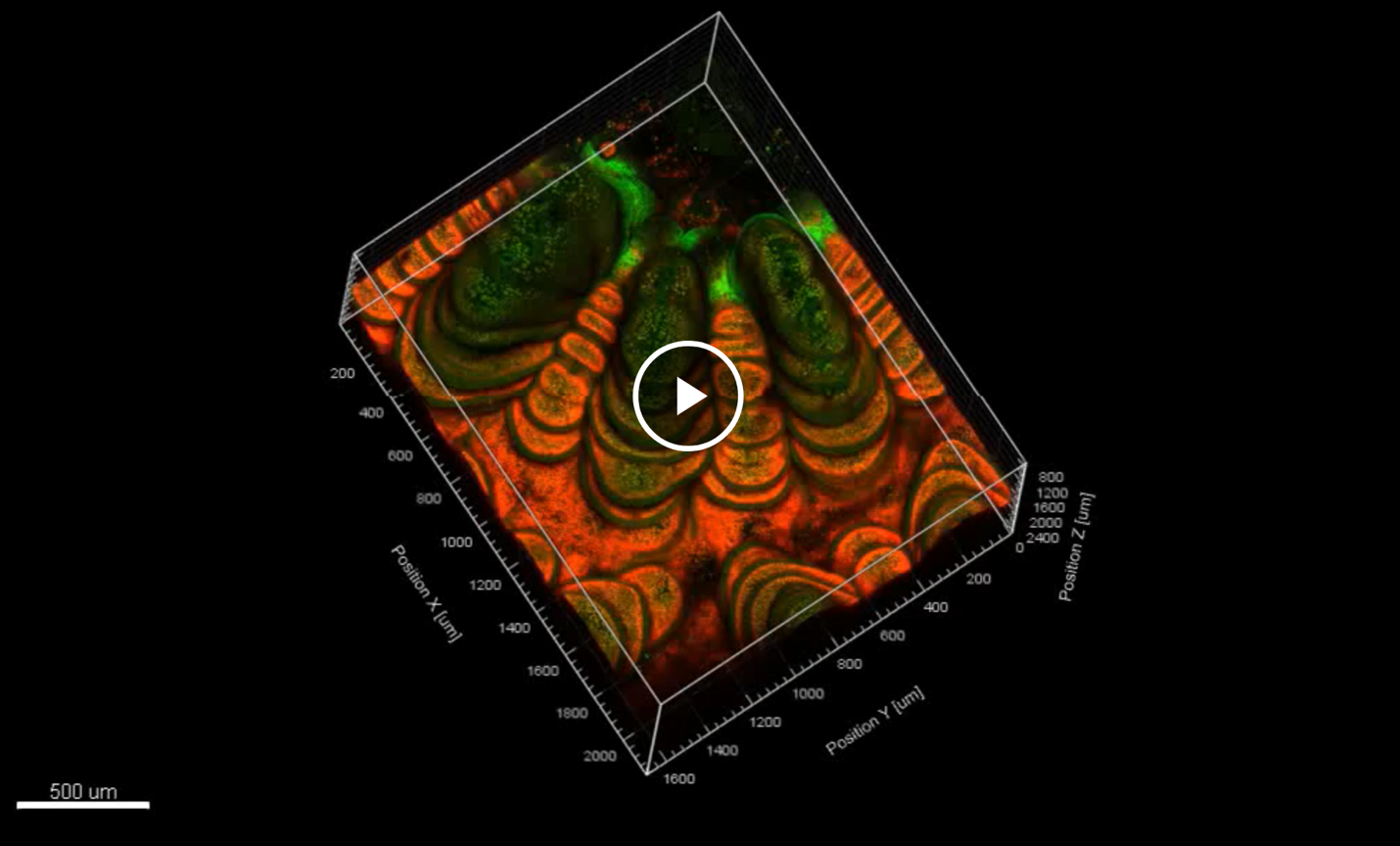


**Supplementary Movie 5. Three-Dimensional Volumetric Quantification of Zooxanthellae and Chromatophores of Representative Segment of a Coral Polyp from *O. faveolata*.** 3D raw data two-photon microscopy image of the coral polyp displayed in the Supplementary Figure 4K. The excitation is 780 nm and the emission captured simultaneously at two band widths for zooxanthellae (pseudo-colored red, 600-700 nm) and chromatophores (pseudo-colored green, 500-550 nm). This movie displays an aerial and profile topographic view from a region of the folded polyp wall of the coral. The regions included in this movie are two tissues overlying the primary and secondary septa. The image is rendered inside a 3D grid with a scale.


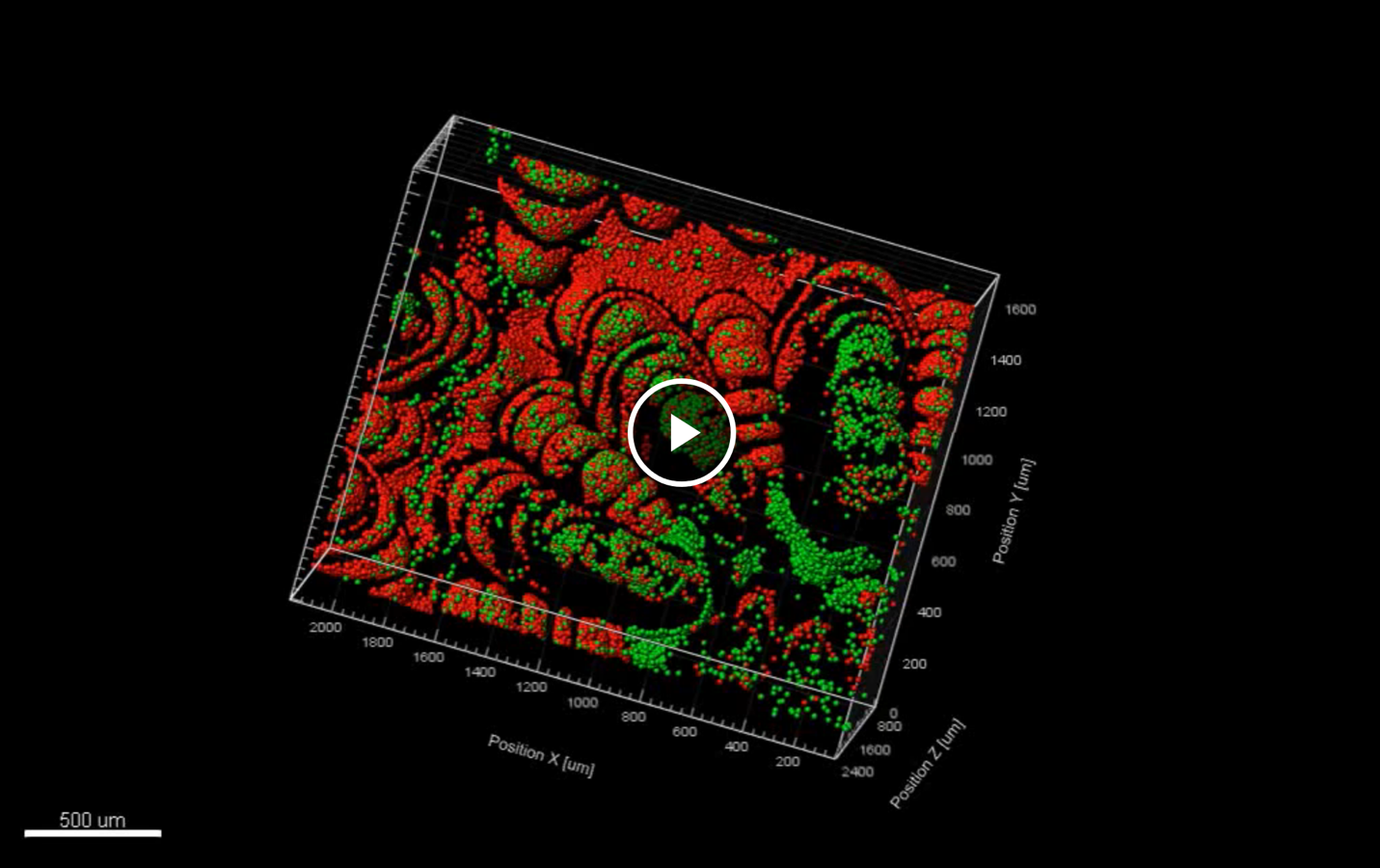


**Supplementary Movie 6. Three-Dimensional Volumetric Quantification of Zooxanthellae and Chromatophores of Representative Segment of a Coral Polyp from *O. faveolata*.** 3D-rendered image of Supplementary Figure 4K and Supplementary Movie 5 after spot quantification by the Imaris 3D-spot Isosurface rendering algorithm. The excitation is 780 nm and the emission captured simultaneously at two band widths for zooxanthellae (pseudo-colored red, 600-700 nm) and chromatophores (pseudo-colored green, 500-550 nm). This movie displays an aerial and profile topographic view from a region of the folded polyp wall of the coral. The regions included in this movie are two tissues overlying the primary and secondary septa. The image is rendered inside a 3D grid with a scale.
